# E3 Ubiquitin Ligase CHIP and NBR1-Mediated Selective Autophagy Protect Additively against Proteotoxicity in Plant Stress Responses

**DOI:** 10.1371/journal.pgen.1004116

**Published:** 2014-01-30

**Authors:** Jie Zhou, Yan Zhang, Jingxia Qi, Yingjin Chi, Baofang Fan, Jing-Quan Yu, Zhixiang Chen

**Affiliations:** 1 Department of Horticulture, Zijingang Campus, Zhejiang University, Hangzhou, China; 2 Department of Botany and Plant Pathology, Purdue University, West Lafayette, Indiana, United States of America; University of Missouri, United States of America

## Abstract

Plant stress responses require both protective measures that reduce or restore stress-inflicted damage to cellular structures and mechanisms that efficiently remove damaged and toxic macromolecules, such as misfolded and damaged proteins. We have recently reported that NBR1, the first identified plant autophagy adaptor with a ubiquitin-association domain, plays a critical role in plant stress tolerance by targeting stress-induced, ubiquitinated protein aggregates for degradation by autophagy. Here we report a comprehensive genetic analysis of CHIP, a chaperone-associated E3 ubiquitin ligase from *Arabidopsis thaliana* implicated in mediating degradation of nonnative proteins by 26S proteasomes. We isolated two *chip* knockout mutants and discovered that they had the same phenotypes as the *nbr1* mutants with compromised tolerance to heat, oxidative and salt stresses and increased accumulation of insoluble proteins under heat stress. To determine their functional interactions, we generated *chip nbr1* double mutants and found them to be further compromised in stress tolerance and in clearance of stress-induced protein aggregates, indicating additive roles of CHIP and NBR1. Furthermore, stress-induced protein aggregates were still ubiquitinated in the *chip* mutants. Through proteomic profiling, we systemically identified heat-induced protein aggregates in the *chip* and *nbr1* single and double mutants. These experiments revealed that highly aggregate-prone proteins such as Rubisco activase and catalases preferentially accumulated in the *nbr1* mutant while a number of light-harvesting complex proteins accumulated at high levels in the *chip* mutant after a relatively short period of heat stress. With extended heat stress, aggregates for a large number of intracellular proteins accumulated in both *chip* and *nbr1* mutants and, to a greater extent, in the *chip nbr1* double mutant. Based on these results, we propose that CHIP and NBR1 mediate two distinct but complementary anti-proteotoxic pathways and protein's propensity to aggregate under stress conditions is one of the critical factors for pathway selection of protein degradation.

## Author Summary

Environmental stresses such as heat cause generation of misfolded and damaged proteins, which are highly toxic and must be efficiently removed. In plants, NBR1, the first isolated autophagy receptor with an ubiquitin-association domain, plays a critical role in plant stress tolerance by targeting ubiquitinated protein aggregates under stress conditions for degradation by autophagy. To study how stress-induced misfolded and damaged proteins are detected and ubiquitinated in plant cells, we analyzed the chaperone-associated E3 ubiquitin ligase CHIP from *Arabidopsis thaliana* for its role in protection against proteotoxicity in plant stress responses. Disruption of *Arabidopsis CHIP* caused increased sensitivity to a spectrum of abiotic stresses as found in the *Arabidopsis nbr1* mutants. Disruption of both *Arabidopsis CHIP* and *NBR1* further compromised plant stress tolerance, indicating that their roles are additive. Furthermore, in the *chip nbr1* double mutant, compromised heat tolerance was associated with increased accumulation of insoluble proteins derived mostly from heat-sensitive but biologically important proteins such as Rubisco activase, catalases and proteins required for protein synthesis and folding. Importantly, stress-induced protein aggregates were still highly ubiquitinated in the *chip* mutants. These results strongly suggest that CHIP and NBR1 function in two distinct but complementary anti-proteotoxic pathways in plant stress responses.

## Introduction

A newly synthesized polypeptide must fold and at times refold into a proper conformation in order to function properly in the cell. However, folding into a proper conformation is a complex process and misfolding is inevitable. In addition, cellular proteins can be damaged by stress conditions such as high temperature, high salt concentrations and reactive molecules. Misfolded and damaged proteins are highly toxic to the cell as they can engage in inappropriate interactions with important cellular components. To monitor, repair or degrade misfolded and damaged proteins, the cell relies on an elaborately regulated protein quality control system, which consists of molecular chaperones such as Hsp70 that promote folding and refolding of nonnative proteins and the protein degradation systems that remove misfolded and damaged proteins [1]. Most soluble misfolded proteins are degraded through the ubiquitin proteasome system (UPS) in which a misfolded protein is ubiquitinated by an enzymatic E1/E2/E3 ubiquitination cascade for targeted destruction by the 26S proteasome. Misfolded and damaged proteins can also be degraded by selective autophagy, which often also relies on ubiquitination for cargo recognition and delivery through such autophagy receptors as P62 and NBR1, which recognize ubiquitinated misfolded and damaged proteins through their ubiquitin association domain [1].

As sessile organisms, plants are constantly exposed to a variety of stress conditions that cause damage to cellular molecules and structures. To survive, plants have evolved complex mechanisms for sensing, responding and adapting to the ever-changing and often stressed environmental conditions. A large number of studies have shown that protein ubiquitination plays a critical role in plant stress responses [2]. Expression of multiple polyubiquitin genes is induced by stress conditions such as high temperatures in plants [3], [4], [5] and overexpression of a single mono-ubiquitin gene increased tolerance of transgenic plants to multiple stresses [6]. Furthermore, mutations of the genes for the 19S regulatory particle subunits of the 26S proteasome reduces plant tolerance to salt, UV radiation and heat shock, indicating that UPS plays a critical role in general plant stress responses [7], [8], [9], [10], [11]. Interestingly, almost all analyzed ubiquitin E3 ligases that regulate plant abiotic stress responses function through modulating the levels of regulatory proteins, mostly transcription factors [2]. By contrast, very little is known about the roles of plant UPS in removing misfolded and damaged proteins that accumulate under stress conditions. This is contrary to the intensive interest and extensive knowledge about cellular pathways for clearance of damaged and toxic macromolecules in mammalian organisms, which are linked with a large number of human diseases including prominent neurodegenerative disorders such as Alzheimer, Parkinson and Huntington's diseases [12], [13].

Autophagy is another evolutionarily conserved major route of protein degradation [1], [14]. Autophagy plays a critical role in plant nutrient recycling and utilization and responses to both biotic and abiotic stresses [15], [16], [17]. Under nutrient starvation, autophagy provides an internal source of nutrients under starvation through nonselective, bulk degradation of cytoplasmic constituents including proteins and organelles. However, autophagy also functions as a quality control mechanism that selectively targets damaged organelles and toxic macromolecules [17], [18]. Selective autophagy is mediated by autophagy receptors/adaptors that recognize specific autophagy substrates [17], [18]. To understand plant selective autophagy, we have recently analyzed *Arabidopsis* NBR1, the first isolated plant autophagy receptor [19]. We have discovered that NBR1 has a selective role in plant tolerance to heat, oxidative, drought and salt stresses but not in age- and darkness-induced senescence and in resistance to necrotrophic pathogens, which also involve autophagy [20]. The compromised heat tolerance of *atg5*, *atg7*, and *nbr1* mutants was associated with increased accumulation of insoluble, detergent-resistant proteins that were highly ubiquitinated under heat stress [20]. NBR1, which contains an ubiquitin-association domain, also accumulated to high levels with an increasing enrichment in the insoluble protein fraction in the autophagy-deficient mutants under heat stress [20]. These results suggest that NBR1-mediated autophagy targets ubiquitinated protein aggregates most likely derived from denatured and damaged proteins generated under stress conditions.

In animal cells, the carboxyl terminus of the Hsc70-interacting protein (CHIP) plays a critical role in protein quality control by ubiquitinating Hsp70-bound misfolded proteins [21], [22]. CHIP acts as both an Hsp70 co-chaperone through its N-terminal tetratricopeptide repeat (TPR) domain and an E3 ubiquitin ligase through the C-terminal U-box domain [23]. If refolding of denatured or damaged proteins assisted by molecular chaperones such as Hsp70 is not successful, CHIP E3 ubiquitin ligase can introduce ubiquitination and thereby target denatured and damaged proteins for degradation by both UPS and autophagy [1]. For example, α-synuclein, a major component of the protein aggregates associated with Parkinson's disease, is degraded by both UPS and autophagy after ubiquitination by CHIP [24], [25], [26]. Overexpression of CHIP decreases aggregation of proteins and cell death associated with chronic neurodegenerative diseases including Parkinson's and Alzheimer [27], [28], [29]. By contrast, deficiency of CHIP in knockout mice decreases longevity associated with accelerated aging phenotypes accompanied by altered protein quality control [30], [31].

*Arabidopsis* CHIP protein is structurally highly similar to animal CHIPs with three tetratricopeptide repeats at the N-terminal side and a U-box domain at the C-terminal side [32]. In addition, *Arabidopsis* CHIP interacts with molecular chaperones such as Hsc70 and has E3 ubiquitin ligase activity *in vitro* [32]. A number of studies have previously reported functional analysis of *Arabidopsis* CHIP in protein turnover and stress responses. CHIP is induced by high cytosolic levels of chloroplast-destined precursor proteins and promotes their degradation by UPS when co-overexpressed with Hsc70-4 in protoplasts [33]. CHIP also interacts with and ubiquitinates chloroplast FtsH and Clp4 proteases and protein phosphatase 2A (PP2A) and the levels of FtsH and Clp4 proteins were reduced but those of PP2A were increased in transgenic plants overexpressing CHIP [34], [35], [36]. CHIP is induced by stress conditions including cold temperature, heat and salt [32]. However, overexpression of *CHIP* in transgenic *Arabidopsis* plants increases sensitivity to both low and high temperatures [32]. The phenotypes of the *CHIP*-overexpressing transgenic plants are unexpected given the positive roles of the E3 ligase in protein quality control. However, even in human cells, when chronically overexpressed, CHIP can affect essential signaling pathways and causes deleterious effects on cellular health [37]. In the present study, we isolated two independent T-DNA knockout mutants for the *CHIP* gene from *Arabidopsis*. The *chip* mutants are normal under normal growth conditions but are hypersensitive to heat, salt and oxidative stresses. To study its functional interaction with NBR1, we generated *chip nbr1* double mutants and provided genetic evidence that CHIP functions additively with NBR1 in plant stress responses. Further analysis including proteomic profiling of stress-induced protein aggregates strongly suggest that CHIP and NBR1 mediate two distinct but complementary anti-proteotoxic pathways in plant stress responses.

## Results

### Identification of *Arabidopsis chip* knockout mutants

**Figure 1:**
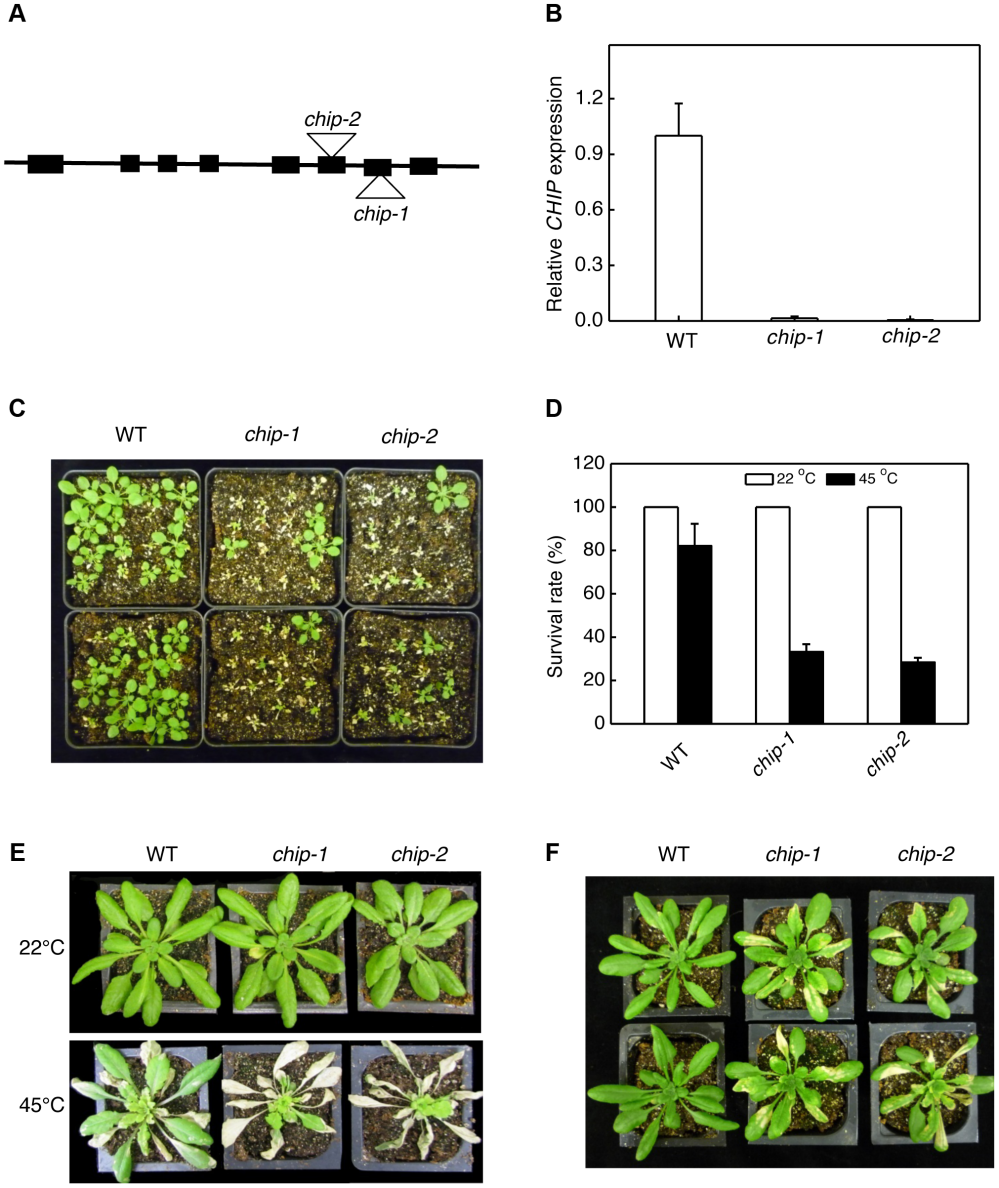
Identification and analysis of *chip* mutants for heat and oxidative stress tolerance. (A) Diagram of the *CHIP* gene and insertion sites. (B) Transcript levels of CHIP in Col-0 wild type (WT) and chip mutants as determined using qRT-PCR. Error bars indicate SE (n = 3). (C) Assays of heat tolerance of young seedlings. Approximately 70 two-weeks old seedlings were placed in a 45°C growth chamber for 9 hours. The heat-treated plants were then moved to a 22°C growth chamber for recovery. The picture was taken at three days after the heat treatment. (D) Survival rates of heat-stressed young seedlings. Approximately 70 two-weeks seedlings were placed in a 45°C growth chamber for 9 hours. The heat-treated plants were then moved to a 22°C growth chamber for recovery. The survival rates were determined at three days after the heat treatment. Error bars indicate SE (n = 3). (E) Assays of heat tolerance of mature plants. Six-weeks old mature plants were placed in a 45°C growth chamber for 9 hours. The heat-treated plants were then moved to a 22°C growth chamber for recovery. The picture was taken at three days after the heat treatment. (F) Assays of tolerance to oxidative stress. Six-weeks old mature plants were sprayed with 20 µM methyl viologen (MV) and kept under light for two days before the picture of representative plants was taken. The experiments were repeated three times with similar results.

To genetically analyze the biological functions of *Arabidopsis* CHIP, we screened T-DNA insertion stocks and identified two independent T-DNA insertion mutants for *Arabidopsis CHIP*. The *chip-1* mutant (Salk_048371) contains a T-DNA insertion in the seventh exon and the *chip-2* mutant (Salk_059253) contains a T-DNA insertion in the sixth exon of the *CHIP* gene (Figure 1A). Quantitative RT-PCR showed that the two mutants had less than 1% of the wild-type level of *CHIP* transcript (Figure 1B), indicating that they are likely null mutants. Both *chip-1* and *chip-2* mutants were normal in growth and development and displayed no detectable morphological phenotypes throughout the entire life cycle when grown under normal growth conditions.

### Increased sensitivity of *chip* mutants to a broad spectrum of abiotic stresses

As a chaperone-associated E3 ubiquitin ligase, animal CHIP proteins ubiquitinate misfolded and damaged proteins and target their degradation by both 26S proteasomes and P62/NBR1-mediated selective autophagy. To examine whether *Arabidopsis* CHIP plays a similar role in plant stress tolerance through ubiquitination of stress-induced protein aggregates in NBR1-mediated selective autophagy, we first analyzed the *chip* mutants for responses to a spectrum of abiotic stresses. For testing heat tolerance, three-weeks-old seedlings of wild type and *chip* mutants were placed in a 45°C growth chamber for 9 hours and scored for survival rates after recovery for 5 days at the room temperature. As shown in Figure 1C & 1D, more than 80% of wild-type seedlings but only about 30% of *chip* mutant seedlings survived after the heat stress. When 5-weeks old mature plants were heat-stressed for 9 hours at 45°C, 70–80% of wild-type leaves but less than 20% of *chip* mutant leaves remained green after 5-day recovery at the room temperature (Figure 1E). These results indicated that the heat tolerance of the *chip* mutants was substantially compromised. To test tolerance to oxidative stress, we sprayed 5-weeks old wild type, *chip-1*, *chip-2* mutants with 20 µM methyl viologen (MV), a reactive oxygen species (ROS)-generating herbicide, and kept the plants under light for two days. For wild-type plants, only old leaves were significantly bleached but more than 90% of leaf areas remained green (Figure 1F). By contrast, more than 50% of leaf tissues of *chip-1* and *chip-2* mutant plants were bleached after MV treatment (Figure 1F). Thus, the *chip* mutant plants were also compromised in tolerance to oxidative stress.

**Figure 2:**
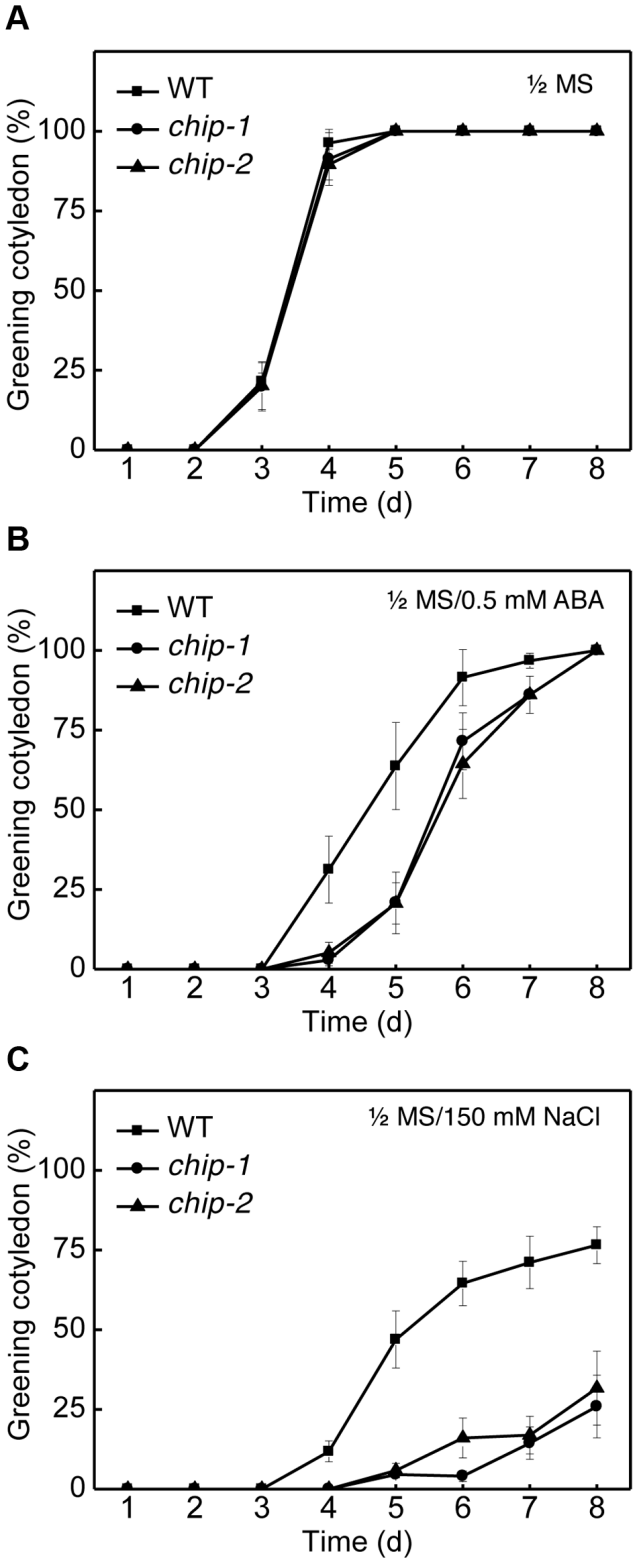
Response of the *chip* mutants to ABA (germination) and salt. Approximately 100 seeds each for Col-0 wild type (WT) and chip mutants were sterilized and sown on 1/2× MS medium (A) or on 1/2× MS medium containing 0.5 µM ABA (B) or 150 mM NaCl (C). Greening cotyledons were scored at the indicated days after sowing. Error bars indicate SE (n = 3).

We also compared the wild type and *chip* mutant plants for responses to abscisic acid (ABA) and salt stress. Wild-type and *chip* mutant seeds were sown on 0.5× Murashige and Skoog (MS) agar medium with or without ABA (0.5 µM) or NaCl (150 mM). Germination rates were determined daily for the following 8 days through scoring of green cotyledons. On the MS medium with no added ABA or NaCl, there was no difference in germination rates between wild type and *chip* mutants, with close to 100% germination after 4 days on the medium (Figure 2A). On the MS medium containing 0.5 µM ABA, however, germination of the *chip* mutants were substantially delayed when compare to that of wild-type (Figure 2B). For example, more than 30% of wild-type seeds but almost no *chip* mutant seeds had green cotyledons after 4 days (Figure 2B). Likewise, more than 60% of wild type but only 20% of *chip* mutants had green cotyledons after 5 days on the ABA-containing MS medium (Figure 2B). After 8 days, however, almost 100% of wild type and *chip* mutant seeds had green cotyledons (Figure 2B). On the MS medium containing 150 mM NaCl, the percentages of wild-type seeds with green cotyledons increased steadily to about 80% after 8 days (Figure 2C). However, only about 20–30% of *chip* mutants had green cotyledons after 8 days on the NaCl-containing medium (Figure 2C). Thus, the *chip* mutants were highly compromised in salt stress.

We also analyzed the responses of the *chip* mutants to the hemibiotrophic bacterial pathogen *Pseudomonas syringae* and the necrotrophic fungal pathogen *Botrytis cinerea* and found the mutants to be normal in resistance to both pathogens based on both symptom development and pathogen growth. Furthermore, we found that the *chip* mutants were normal in age- and dark-induced senescence. Thus, the *chip* mutants appeared to be compromised specifically in tolerance to abiotic stresses and ABA.

### Accumulation of heat-induced protein aggregates in *chip* mutants

**Figure 3:**
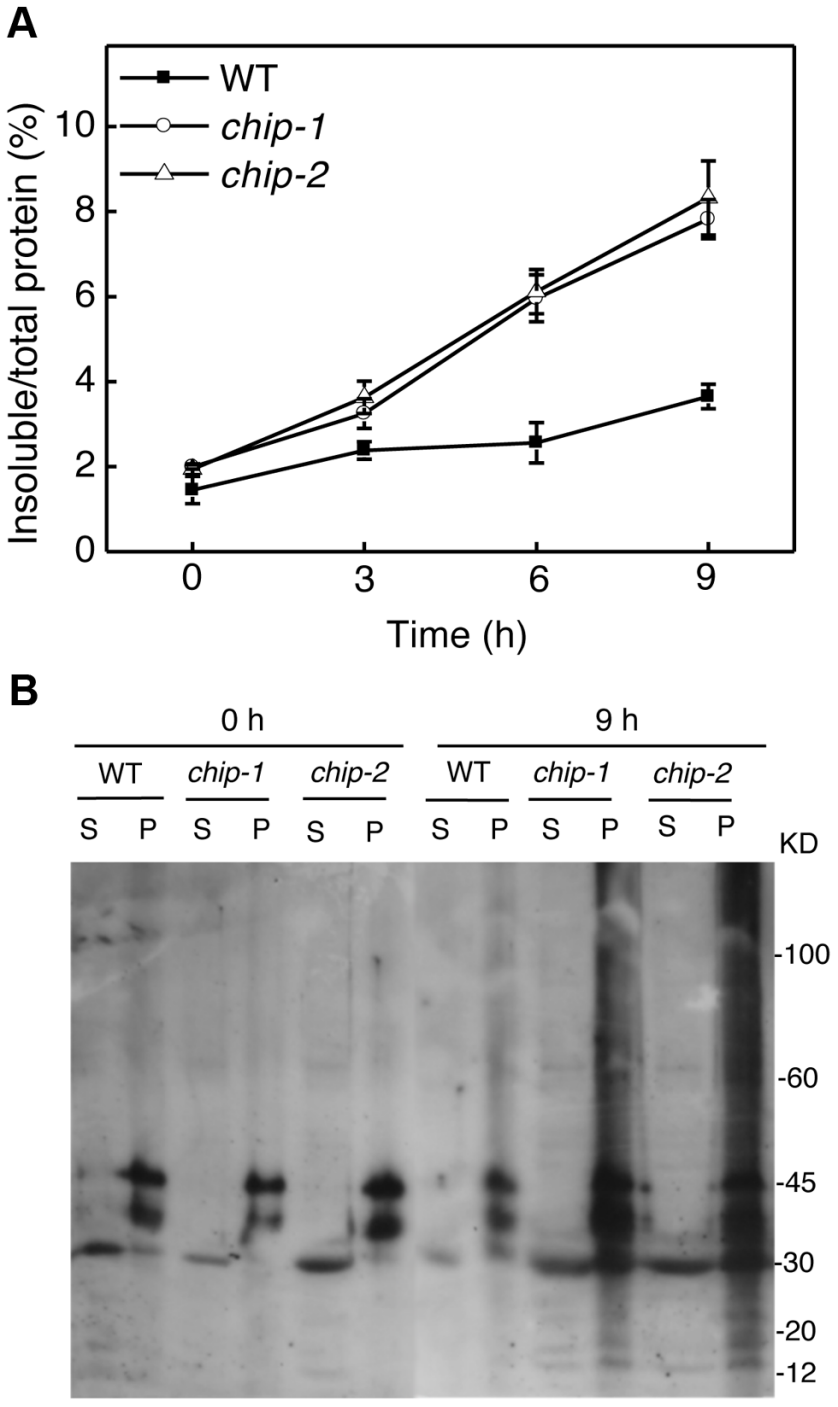
Increased accumulation of insoluble ubiquitinated proteins in the *chip* mutants under heat stress. (A) Accumulation of insoluble proteins. Leaf tissues from wild-type (WT) and *chip* mutants collected at indicated hours (h) at 45°C for preparation of total, soluble and insoluble proteins as described in Materials and Methods. Total proteins in the starting homogenates and insoluble proteins in the last pellets were determined for the percentages of insoluble proteins to total proteins were calculated. (B) Ubiquitination of insoluble protein aggregates in the *chip* mutants under heat stress. Proteins from the first supernatants (S) and last pellets (P) were subjected to SDS-PAGES and probed with anti-ubiquitin monoclonal antibody. The experiment was repeated three times with similar results.

The compromised phenotypes in tolerance to a spectrum of abiotic stresses but normal phenotypes in disease resistance and senescence of the *chip* mutants were strikingly similar to those shown by the *nbr1* mutants [20], supporting that the chaperone-associated E3 ubiquitin ligase might function in the same pathway as NBR1 in plant stress responses. In the *nbr1* mutants, compromised heat tolerance was associated with accumulation of insoluble, detergent-resistant protein aggregates that are most likely derived from heat-denatured or damaged proteins [20]. To determine whether the *chip* mutants also shared this phenotype with the *nbr1* mutants, we compared wild type with the *chip* mutants for the levels of insoluble, detergent-resistant protein aggregates during the 9-hour heat treatment. The plants were placed in a 45°C growth chamber and leaves were collected at various time points for isolation of both total and insoluble proteins. As shown in Figure 3A, insoluble proteins as percentages of total proteins in wild-type plants increased only slightly, from 1.6% to about 3%, after 9-hour heat stress (Figure 3A). By contrast, in both *chip-1* and *chip-2* mutants, insoluble proteins increased as percentages of total proteins from 2% to more than 8% after 9 hours at 45°C (Figure 3A). As a result, the levels of insoluble protein aggregates in the *chip-1* and *chip-2* mutants were about three times of those in wild type after 9-hour heat stress (Figure 3A). Thus, as in *nbr1* mutants, compromised heat tolerance in the *chip* mutants was associated with increased accumulation of heat-induced insoluble protein aggregates.

### Additive phenotypes of the *chip* and *nbr1* mutants in heat tolerance

**Figure 4:**
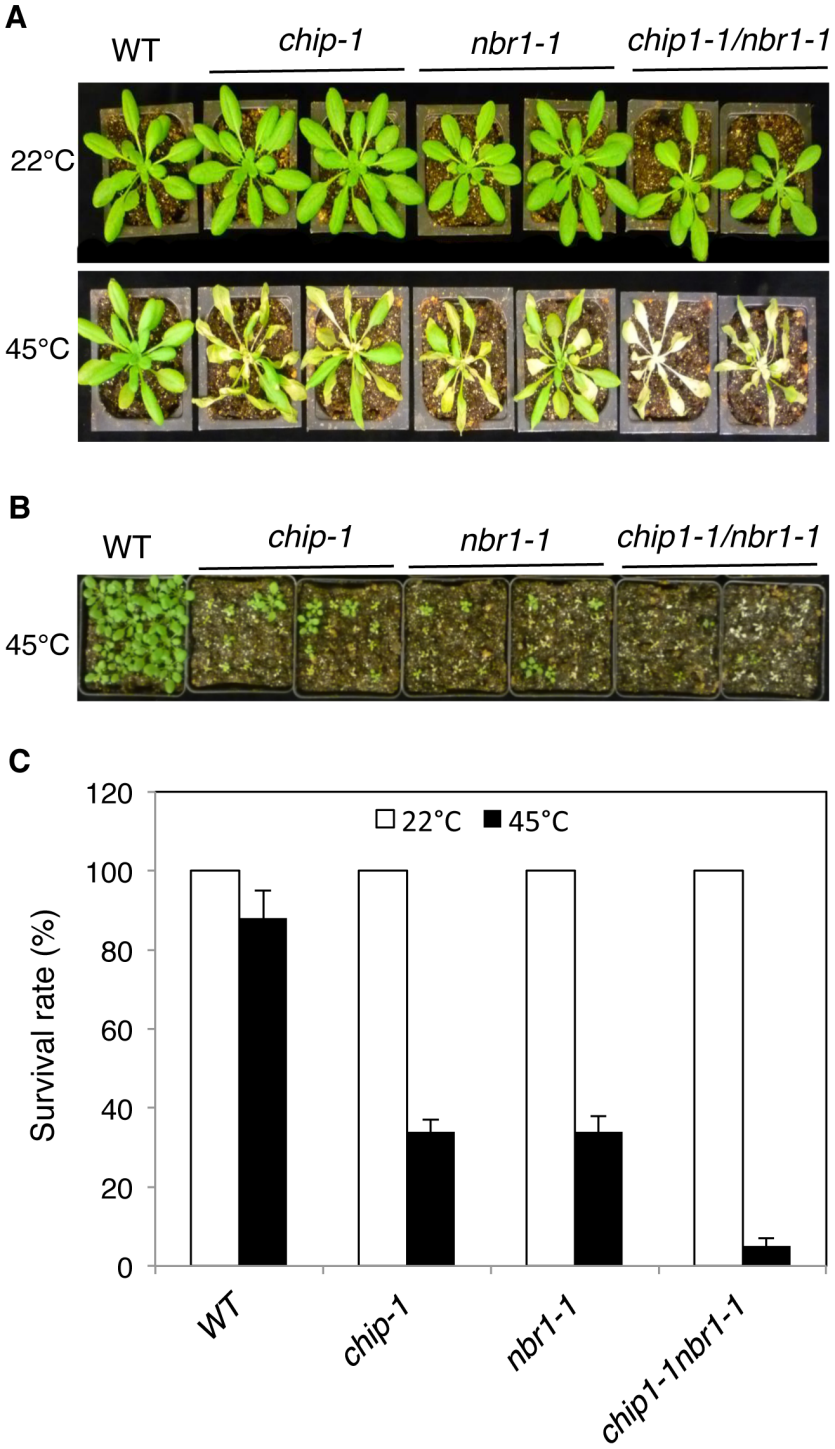
Phenotypic analysis of the *chip nbr1* double mutants in heat tolerance. (A) Assays of heat tolerance of mature plants. Six-weeks old Col-0 wild type (WT), *chip-1*, and *nbr1-1* single mutants and *chip-1 nbr1-1* double mutant plants were placed in a 45°C growth chamber for 9 hours. The heat-treated plants were then moved to a 22°C growth chamber for recovery. The picture was taken at three days after the heat treatment. (B) Assays of heat tolerance of young seedlings. Approximately 70 two-weeks old seedlings were placed in a 45°C growth chamber for 9 hours. The heat-treated plants were then moved to a 22°C growth chamber for recovery. The picture was taken at three days after the heat treatment. (C) Survival rates of heat-stressed young seedlings. Approximately 70 two-weeks seedlings were placed in a 45°C growth chamber for 9 hours. The heat-treated plants were then moved to a 22°C growth chamber for recovery. The survival rates were determined at three days after the heat treatment. Error bars indicate SE (n = 3).

**Figure 5:**
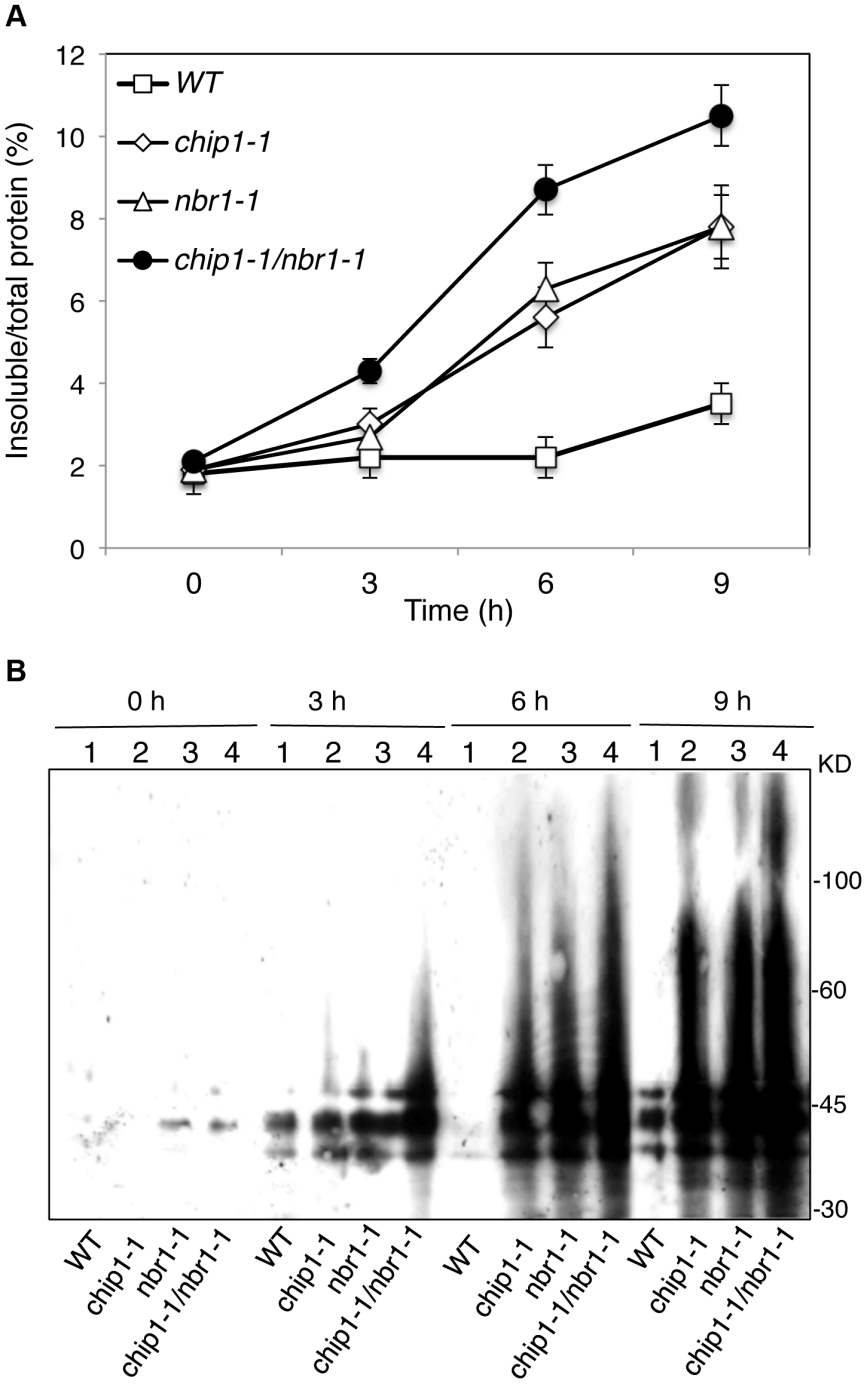
Accumulation and ubiquitination of insoluble proteins in the *chip*, *nbr1* single and double mutants under heat stress. (A) Accumulation of insoluble proteins. Leaf tissues from wild-type (WT), *chip, nbr1* single and double mutants collected at indicated hours (h) under 45°C for preparation of total, soluble and insoluble proteins as described in Materials and Methods. Total proteins in the starting homogenates and insoluble proteins in the last pellets were determined for the percentages of insoluble proteins to total proteins were calculated. (B) Ubiquitination of insoluble protein aggregates in wild type (WT), *chip, nbr1* single and double mutants collected at indicated hours (h) under heat stress. Proteins from the last pellets were subjected to SDS-PAGES and probed with anti-ubiquitin monoclonal antibody. The experiment was repeated three times with similar results.

The strikingly similar phenotypes of the *chip* and *nbr1* mutants in compromised stress tolerance and in accumulation of stress-induced protein aggregates strongly suggested that *Arabidopsis* CHIP and NBR1 have similar roles in plant stress responses. To analyze genetically the functional relationship between CHIP and NBR1, we generated the *chip-1 nbr1-1* double mutant and compared it with its parental *chip* and *nbr1* single mutants for heat tolerance. When 5-weeks old mature plants were heat-stressed for 9 hours at 45°C, more than 80% of wild-type leaves and 20–30% of *chip* and *nbr1* single mutant leaves remained green after 5-day recovery at room temperature (Figure 4A). By contrast, no green leaves from the mature *chip nbr1* double mutants were detected after 5-day recovery at room temperature following 9-hour heat stress (Figure 4A). We also placed two-weeks-old seedlings of wild type and mutants in a 45°C growth chamber for 9 hours and scored for survival rates after recovery for 5 days at room temperature. More than 80% of wild-type seedlings and about 30% of *chip* and *nbr1* single mutant seedlings survived after the heat stress (Figure 4B & 4C). By contrast, only about 5% of the *chip nbr1* double mutant seedlings survived after the heat stress (Figure 4B & 4C). Thus, assays of both mature plants and seedlings indicated that the *chip nbr1* double mutants were more compromised in heat tolerance than the parental *chip* and *nbr1* single mutants. In addition, we compared the *chip nbr1* double mutant with the *chip* and *nbr1* single mutants for the levels of insoluble, detergent-resistant protein aggregates during the 9-hour heat treatment. At the three time points assayed (3, 6 and 9 hours), the levels of insoluble protein aggregates in the *chip nbr1* double mutant were about 30–40% higher than those in the *chip* and *nbr1* single mutants (Figure 5A). This result supported that the roles of CHIP and NBR1 in plant heat tolerance were additive.

### Normal ubiquitination of stress-induced protein aggregates in the *chip* mutants

Previously it has been shown that CHIP E3 ubiquitin ligase and its interacting Hsc70 mediate degradation of plastid-destined precursor proteins by the 26S proteasomes to prevent cytosolic precursor accumulation [33]. With the important role of CHIP in plant tolerance to abiotic stresses, we examined whether CHIP is involved in ubiquitination of heat-induced protein aggregates, which accumulated in the *chip* mutants under heat stress. We isolated both soluble and insoluble proteins from the wild type and *chip* mutants collected before and after 9-hour heat stress. The proteins were fractionated by SDS-PAGE and analyzed for ubiquitinated proteins using an anti-ubiquitin monoclonal antibody. As shown in Figure 3B, we observed similar levels of ubiquitinated proteins in the soluble fractions in these plants with or without heat stress. In the insoluble fractions, we observed similar levels of ubiquitinated proteins in the *chip-1* and *chip-2* mutants and in wild type before heat treatment (Figure 3B). However, after 9-hour heat stress, we observed a drastic increase in the levels of ubiquitinated proteins in the *chip-1* and *chip-2* mutants but not in the wild-type plants (Figure 3B). Thus, stress-induced insoluble proteins from heat-stressed *chip* mutants are still highly ubiquitinated.

To examine combined effects of CHIP and NBR1, we also compared the *chip nbr1* double mutant with its parental *chip* and *nbr1* single mutants for the changes in the levels of insoluble ubiquitinated proteins after 3-, 6-, and 9-hour heat treatment. As shown in Figure 5B, there were little or slight increases in the levels of ubiquitinated insoluble proteins in the wild-type plants during the 9-hour heat stress. On the other hand, ubiquitinated insoluble proteins increased steadily and to similar levels in the *chip* and *nbr1* single mutants with time of heat stress (Figure 5B). In the *chip nbr1* double mutant plants, ubiquitinated insoluble proteins increased to even higher levels than those in their parental *chip* and *nbr1* single mutants during the 9-hour heat treatment (Figure 5B). These results demonstrated the additive roles of CHIP and NBR1 in protection against proteotoxicity in plant stress responses and provided further evidence that CHIP is dispensable for ubiquitination of heat-induced protein aggregates targeted by NBR1-mediated selective autophagy.

### Proteomic profiling of heat-induced insoluble protein aggregates

**Figure 6:**
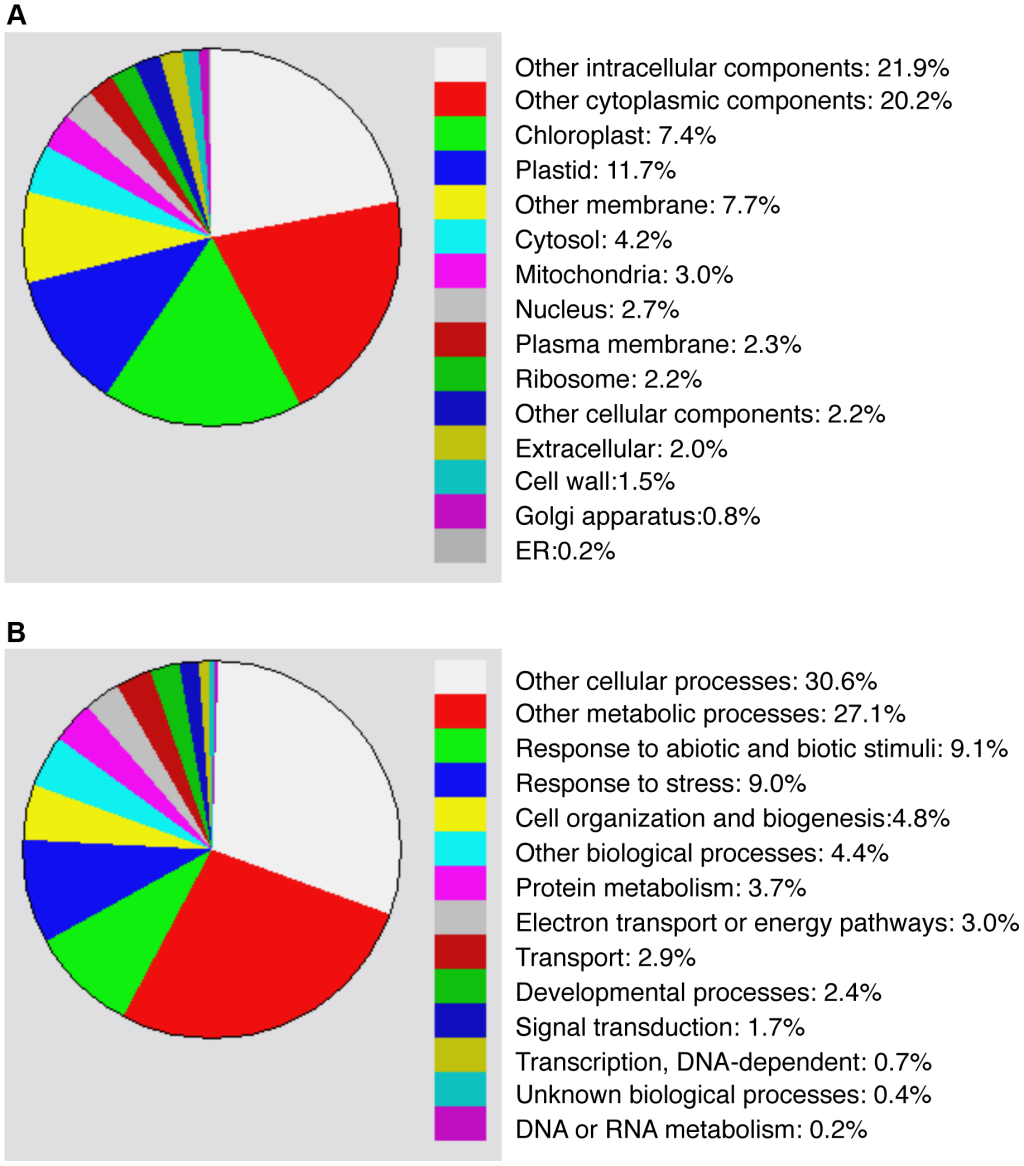
Functional categorization of heat-induced insoluble proteins. Proteins identified from proteomic profiling of protein aggregates accumulated in heat-stressed *chip* and *nbr1* mutants were grouped using the GO program based two gene product properties: Cellular component (A) and biological process (B).

Compromised heat tolerance of *chip* and *nbr1* single and double mutants was associated with increased accumulation of insoluble ubiquitinated protein aggregates under heat stress (Figures 5). To identify proteins targeted by CHIP- and NBR1-mediated degradation pathways under heat stress, we isolated insoluble protein aggregates from wild type, *chip* and *nbr1* single and double mutant plants after 6 and 9-hour heat stress and subjected them to shotgun LC-MS/MS profiling. From the proteomic profiling of the four genotypes with two time points of heat stress, more than 16,000 tryptic peptide sequences were obtained, which belong to 440 non-redundant candidate proteins (Tables S1 & S2). Because a substantial percentage of the identified peptides matched several members of the same protein family, more than 554 proteins were identified (Tables S1 & S2). Functional categorization of the identified proteins showed that almost 20% of them are components of chloroplasts/plastids but less than 3% are associated with each of the other major organelles including nucleus, mitochondria, Golgi apparatus and endoplasmic reticulum (Figure 6A). Intriguingly, almost 20% of identified proteins are involved in responses to biotic and abiotic stimuli or other stress signals (Figure 6B), making it one of the largest categories for important biological processes in plants.

To investigate differential accumulation of aggregated proteins in the *chip* and *nbr1* mutants, we estimated the relative abundance of aggregated proteins by normalizing the numbers of detected peptides to the amount of total proteins from which aggregated proteins were isolated (Tables S1 & S2). Survey of the most abundant protein aggregates based on the normalized peptide numbers revealed both expected and surprising results. As expected, a large percentage of heat-induced protein aggregates are chloroplast/plastid proteins but their relative abundances in the aggregates were not totally correlated with their relative levels in leaf tissues. For example, Rubisco proteins are the most abundant proteins in leaf tissues but were not the most abundant aggregated proteins after 6-hour heat stress (Table S1). Rubisco activase was one of the most abundant aggregated proteins accumulated in heat-stressed *chip nbr1* mutant plants (Tables S1 & S2). CAT3 and CAT2, two major leaf catalase isoforms in *Arabidopsis* [38], were also present abundantly as aggregated proteins (Tables S1 & S2). Other abundant protein aggregates accumulated in heat-stressed plants include a number of chloroplast-localized light-harvesting complex proteins (Tables S1 & S2). Interestingly, a substantial number of abundant aggregated proteins are involved in protein synthesis, folding/refolding and maturation. These proteins included translation initiation and elongation factors, cyclophilin-type peptidyl-protyl *cis-trans* isomerases and heat shock proteins (Tables S1 & S2).

**Figure 7:**
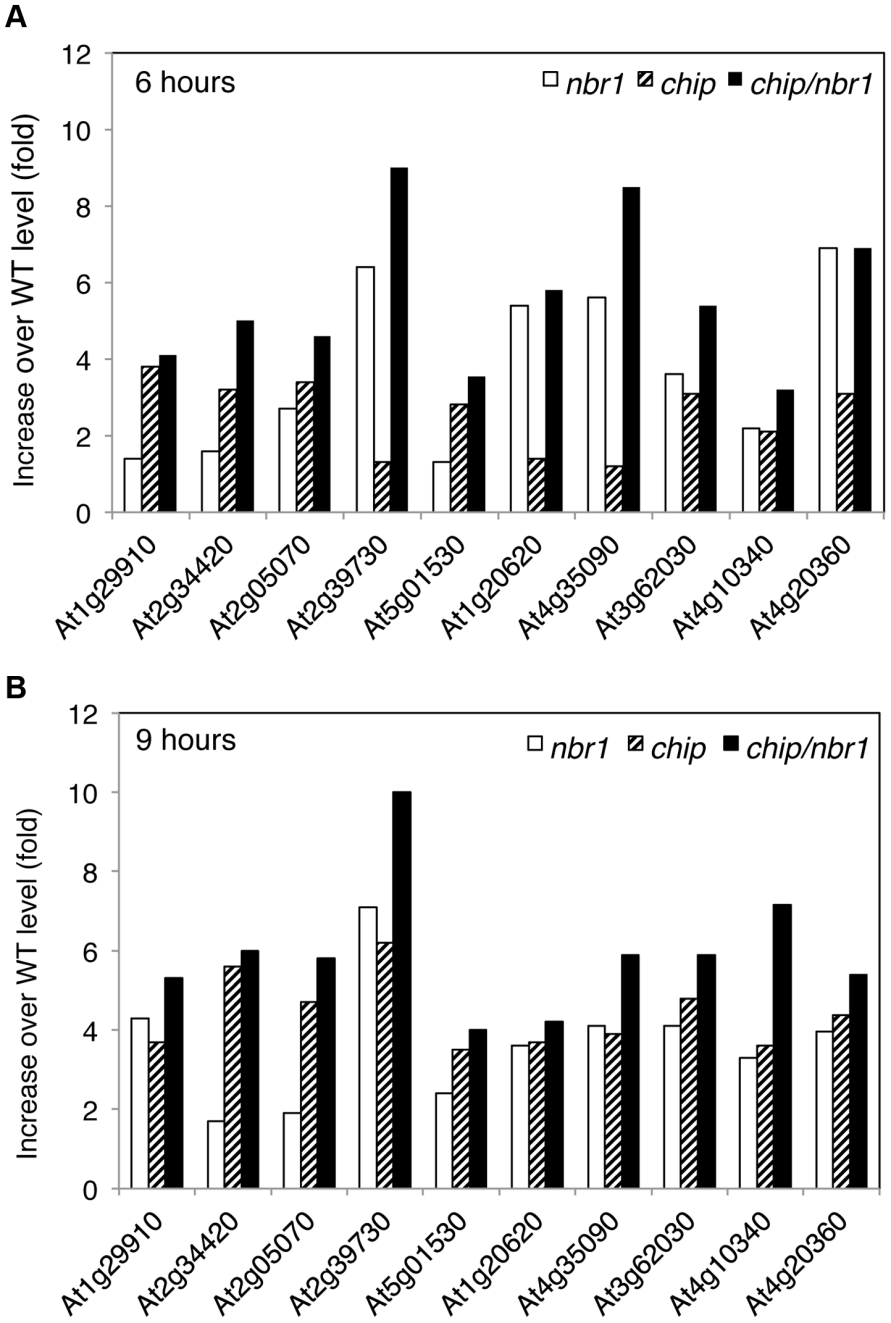
Differential accumulation of heat-induced insoluble proteins in the *chip* and *nbr1* mutants. The relative abundances of the 10 proteins in the insoluble fraction of heat-stressed wild-type and mutant plants were estimated from their detected peptide numbers after normalizing them to the same amount for total protein (0.1 mg) used for isolation of insoluble proteins. Increased accumulation of an aggregated protein in the mutants after 6 (A) and 9 (B) hours of heat stress was determined by calculating the ratio of the normalized peptide number in the mutants to that in wild type. At1g29910, LHCB1; At2g34420, LHCB1B2; At2g05070, LHCB2; At2g39730, RCA; At5g01530, LHCB4; At1g20620, CAT3; At4g35090, CAT2; At3g62030, ROC4; At4g10340, LHCB5; At4g20360, RABE1B.

We also estimated heat-induced enrichment of aggregated proteins in the *chip* and *nbr1* mutants by calculating the ratio of their normalized peptide numbers to those in the wild type. As shown in Figure 7A, among the 10 most abundant aggregated proteins a number of them differentially accumulated in the *chip* or *nbr1* mutants after 6-hour heat stress. For example, the abundances of protein aggregates with peptide sequences matching several highly similar plastid light-harvesting complex (LHC) proteins including those of At1g29910 and At2g34420 were 2 to 3 times higher in the *chip* mutant than in the *nbr1* mutant (Figure 7A). On the other hand, the levels of protein aggregates for Rubisco activase (RCA, At2g39730), CAT3 (At1g20620) and CAT2 (At4g35090) in the *nbr1* mutant were 3 to 5 times higher than those in the *chip* mutant (Figure 7A). Survey of all detected aggregated proteins also showed a substantial percentage of them accumulating differentially in the two mutants after 6-hour heat stress (Tables S1 & S2). Intriguingly, after 9-hour heat stress, relative abundances of Rubisco activase and CAT proteins became similar in the two mutants (Figure 7B). Consistent with the heat sensitive phenotypes, the abundances of protein aggregates in the *chip nbr1* double mutant were generally higher than those in the *chip* and *nbr1* single mutants after both 6- and 9-hour heat stress (Figure 7; Tables S1 & S2).

**Figure 8:**
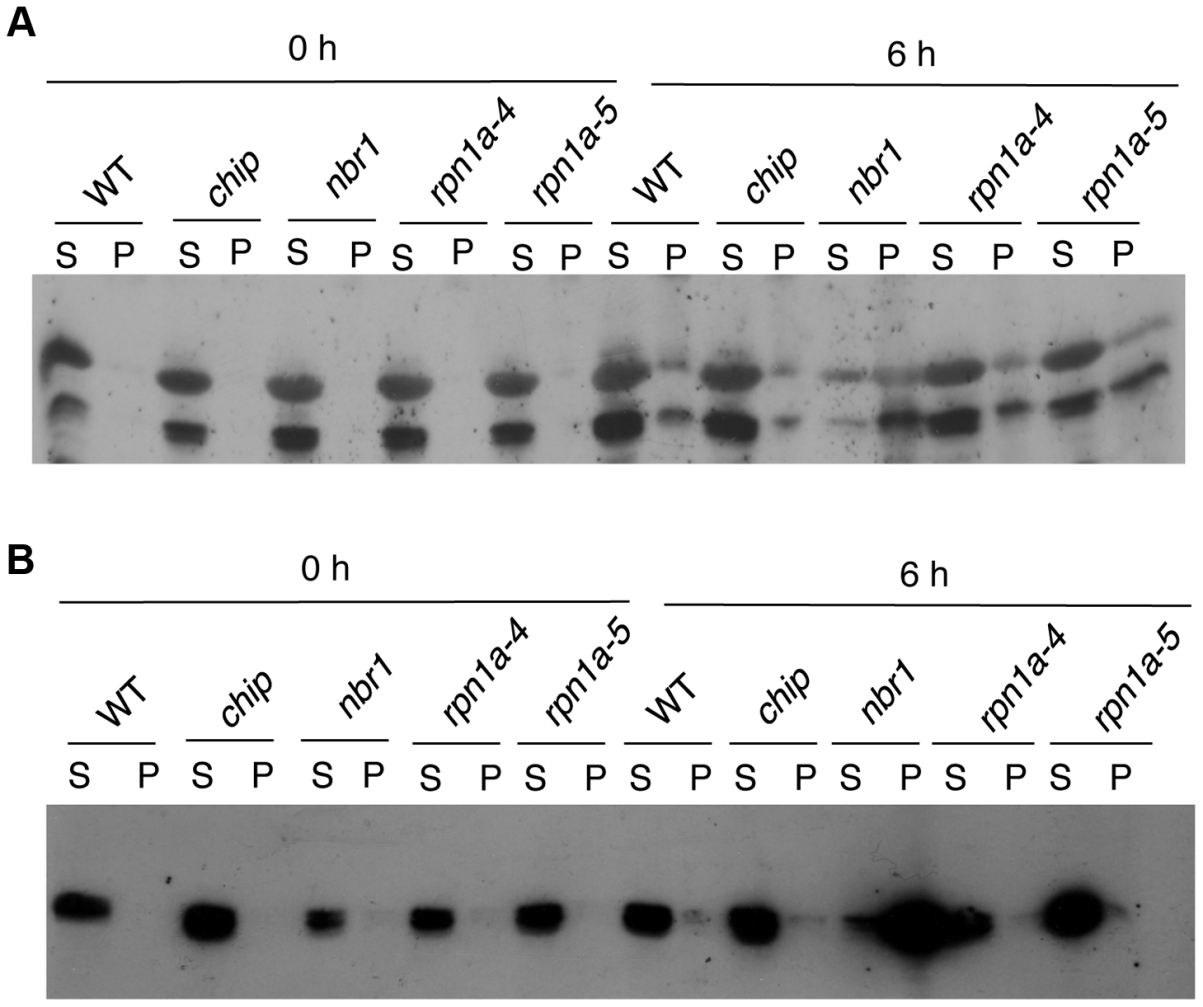
Changes in RCA and catalase proteins in response to heat stress. Proteins from the first supernatants (S) and last pellets (P) isolated from wild type (WT) and *chip*, *nbr1* and *rpn1a* mutants collected at indicated hours (h) under heat stress were subjected to SDS-PAGES and probed with anti-RCA (A) or anti-catalase (B) monoclonal antibody.

To confirm the differential accumulation of protein aggregates from the proteomic profiling, we analyzed the accumulation of RCR and CAT protein aggregates in the *chip* and *nbr1* mutants after 0- and 6-hour heat stress using western blotting. In the western blotting, we included two T-DNA insertion mutants (*rpn1a-4* and *rpn1a-5*) for the *Arabidopsis* 26S proteasome subunit RPN1a, which are also sensitive to heat stress [39]. When both soluble and insoluble proteins were probed with a previously generated RCA antibody [40], two RCA isoforms of 43 and 47 kD arising from mRNA alternative splicing were detected (Figure 8A). Without heat stress, high levels of RCA proteins were detected in the soluble fraction but little RCA proteins were present in the insoluble fractions (Figure 8A). After 6-hour heat stress, high levels of RCA proteins were still present in the soluble fraction of wild type, *chip* and *rpn1a* mutants, although a significant level of the proteins was also detected in the insoluble fraction (Figure 8A). In the *nbr1* mutant, on the other hand, the soluble RCA was reduced while insoluble RCA, particularly the 43-kD isoform, was substantially increased (Figure 8A). Thus, insoluble RCA accumulated to a higher level in the *nbr1* mutant than in the *chip* and *rpn1a* mutants. Likewise, using a catalase monoclonal antibody that recognize both *Arabidopsis* CAT2 and CAT3 (Figure S1), we observed that after 6-hour heat stress, insoluble catalase proteins accumulated at high levels in the *nbr1* mutant but not in wild type, *chip* or *rpn1a* mutants (Figure 8B).

### Aggregation and ubiquitination of catalases under heat stress

**Figure 9:**
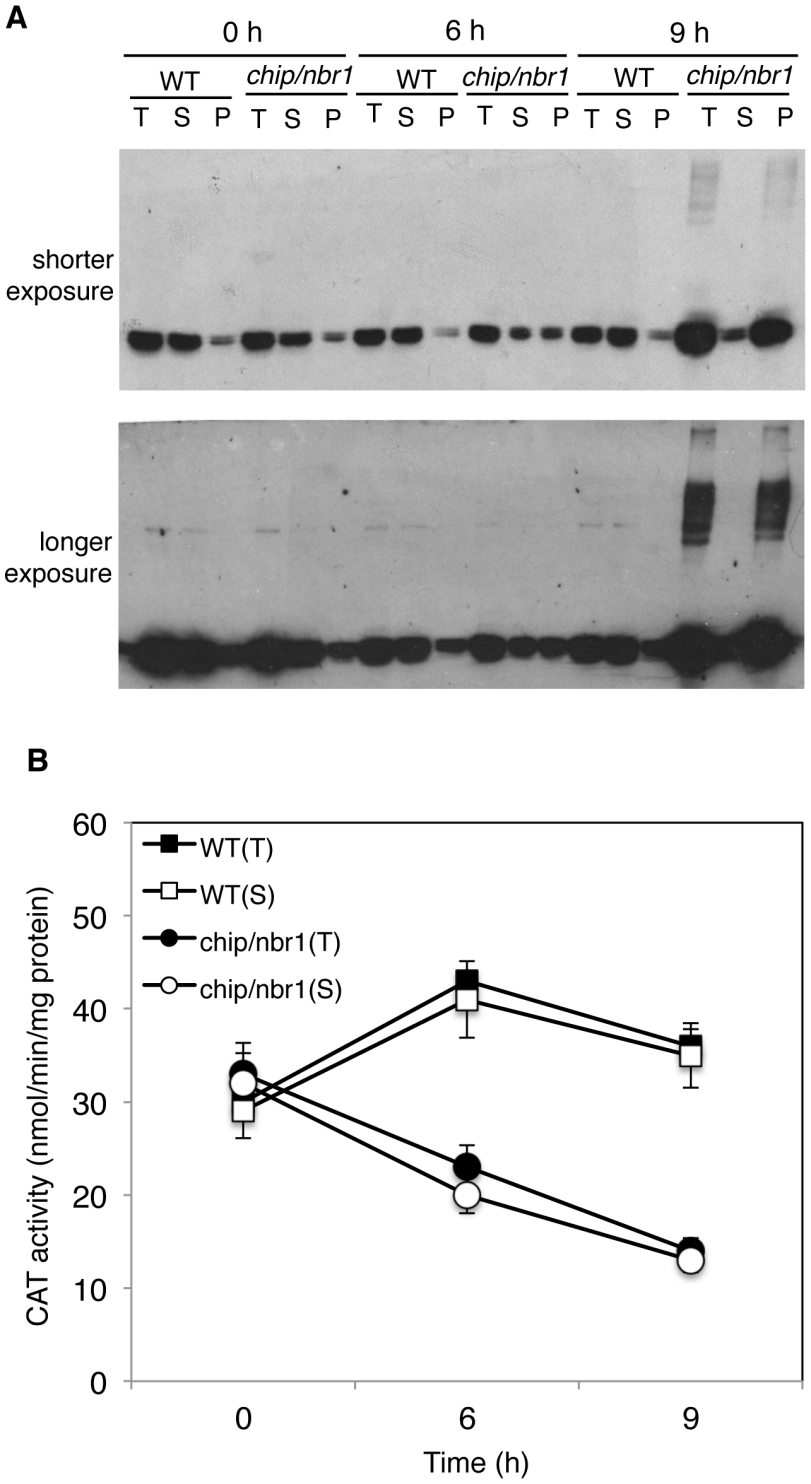
Changes in catalase proteins and activity in response to heat stress. (A) Catalase proteins in wild type (WT) and *chip, nbr1* double mutant collected at indicated hours (h) under heat stress. Total proteins (T), proteins from the first supernatants (S) and last pellets (P) were subjected to SDS-PAGES and probed with anti-catalase monoclonal antibody 3B6. Both a shorter (upper panel) and a longer (lower panel) exposure of the blot were shown so that the relative levels of both low and high molecular weight catalase proteins could be better visualized. (B) Catalase activity in wild type (WT) and *chip, nbr1* double mutant collected at indicated hours (h) under heat stress. Total proteins (T) and proteins from the first supernatants (S) were assayed for catalase activity using a spectrophotometric procedure. Error bars indicate SE (n = 3).

Catalases are antioxidant enzymes with a crucial role in cellular responses to oxidative stress, which are linked with a wide variety of biotic and abiotic stresses including heat stress [41]. *Arabidopsis* CAT3 and CAT2 are two major catalase isoforms in *Arabidopsis* leaves [38] and their abundant presence as aggregated forms in heat-stressed *chip nbr1* mutant plants suggested a possible mechanistic link between stress-induced oxidative stresses and protein quality control. To examine the connection between heat-induced proteotoxic and oxidative stresses, we compared wild type and the *chip nbr1* double mutant for the changes of both the catalase proteins and activity. Before heat stress, wild type and *chip nbr1* mutant plants had similar levels of catalase proteins, which were mostly soluble (Figure 9A). After 6- and 9-hour heat treatment, a majority of catalases remained soluble and no major increases in catalase proteins were observed in wild-type plants (Figure 9A). On the other hand, heat stress increased total catalase protein levels in the *chip nbr1* mutant but a majority of these catalase proteins existed as insoluble proteins (Figure 9A). Importantly, despite higher levels of total catalase proteins, heat-stressed *chip nbr1* mutant plants had lower levels of soluble catalase proteins than heat-stressed wild-type plants (Figure 9A). Furthermore, a majority of aggregated catalases accumulated in heat-stressed *chip nbr1* mutant plants migrated normally on SDS-PAGES when compared to soluble catalase proteins (Figure 9A), indicating that they were not ubiquitinated. A significant fraction of the insoluble catalase aggregates in the *chip nbr1* mutant plants were present as high molecular weight proteins, most likely due to polyubiquitination (Figure 9A).

To determine whether aggregated catalase proteins were catalytically active, we first attempted to measure directly the catalase activity of the insoluble protein fractions but found the assays to be difficult once the protein aggregates were pelleted after centrifugation. Therefore, we used an indirect approach by comparing the catalase activities of the total protein fractions with those of the soluble protein fractions. As shown in Figure 9B, the total and soluble catalase activities in wild-type plants were very similar before or after heat treatment, which were expected given that catalase proteins in wild type remained mostly soluble after heat treatment (Figure 9A). In heat-stressed *chip nbr1* mutant plants, the catalase activities from the total and soluble protein fractions were also very similar (Figure 9B) even though the levels of catalase proteins in the total protein factions were much higher than those in the soluble protein factions (Figure 9A). This result indicated that only the soluble catalases were catalytically active. Consistent with this interpretation, the total catalase activities increased slightly in heat-treated wild-type plants but reduced substantially in heat-treated *chip nbr1* mutant plants. After 9-hour heat treatment, the catalase activities in the *chip nbr1* mutant plants were only about 35% of those in the wild-type plants. In both wild type and *chip nbr1* mutant, the levels of catalase activities were closely correlated with the levels of soluble catalase proteins (Figure 9). Thus, reduced degradation of denatured catalase aggregates in the *chip nbr1* mutant plants not only led to increased accumulation of catalase protein aggregates but also caused reduction of soluble, active catalase proteins.

### Disruption of *CHIP* promoted stress-induced autophagy

**Figure 10:**
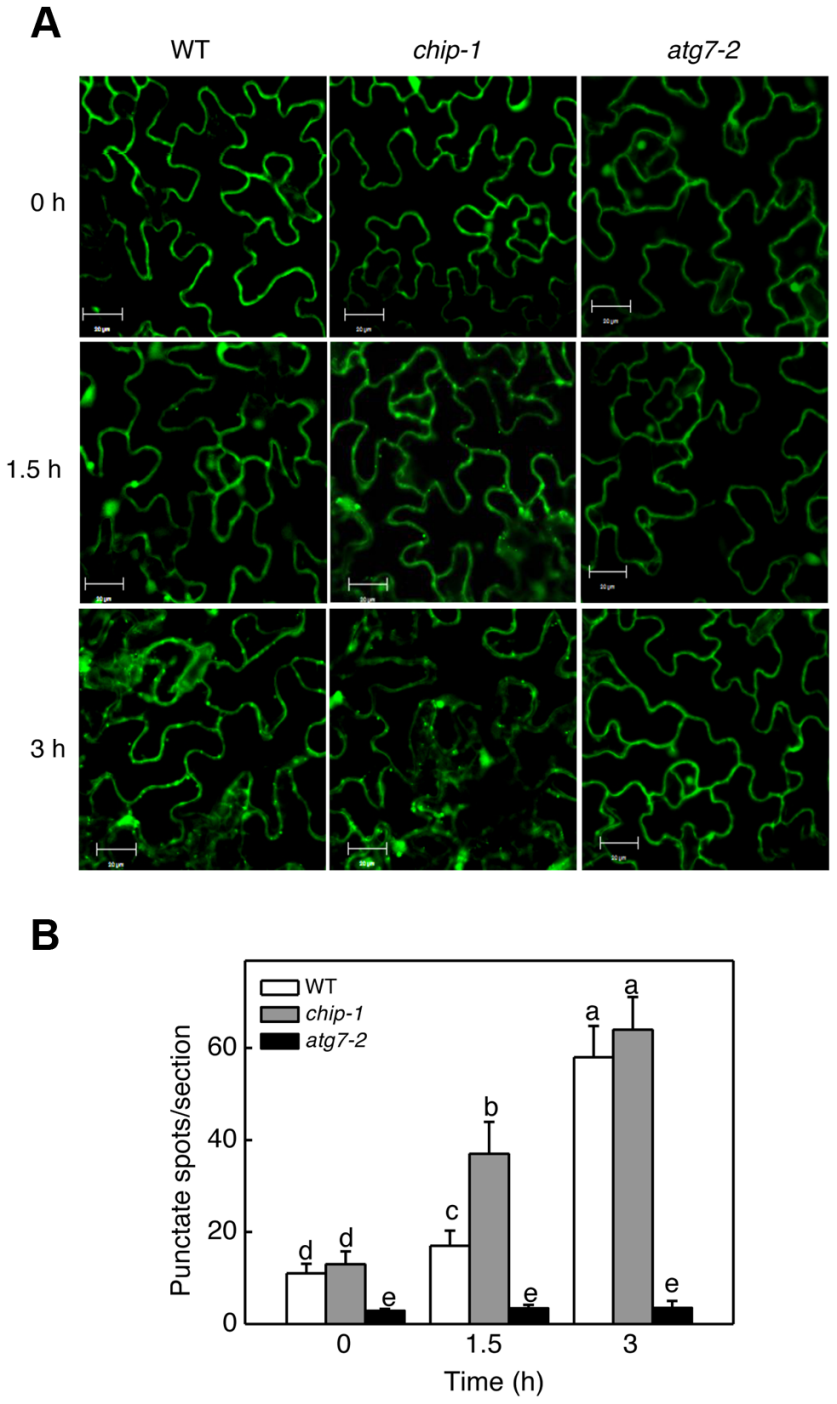
Determination of accumulation of autophagosomes using GFP-ATG8a. (A) Five-weeks old transgenic wild-type Col-0 (WT), *chip-1* and *atg7-2* mutant plants expressing *GFP-ATG8a* were treated with (45°C) or without (22°C) heat shock for indicated hours (h) and then placed at room temperature for 0.5 h. The leaves were visualized by fluorescence confocal microscopy of GFP signal. (B) Numbers of punctate GFP-ATG8a spots representing autophagosomes per 10,000 µm^2^ section. Means and SE were calculated from three experiments. According to Duncan's multiple range test (P = 0.05), means do not differ significantly if they are indicated with the same letter. Bar = 20 µm.

The additive phenotypes and the differential accumulation of aggregated proteins in the *chip* and *nbr1* mutants suggest that CHIP and NBR1 participate in two distinct pathways in degrading misfolded and damaged proteins under stress conditions. This interpretation is consistent with the normal ubiquitination of NBR1-targeted protein aggregates in the *chip* mutants (Figures 3B & 5B). However, since CHIP- and NBR1-mediated anti-proteotoxic pathways contribute to the same process of removing misfolded and damaged proteins under stress conditions, they may be coordinated not only in functions but also in regulation. To test this, we examined the effect of CHIP deficiency on heat-induced autophagy by comparing wild type and *chip* mutants for heat-induced autophagosome formation and autophagy gene expression. We examined the effect of heat stress on induction of autophagosome accumulation using green fluorescent protein (GFP)-tagged ATG8a, which is associated with autophagosomes and has been used as a marker of autophagosomes in *Arabidopsis* [42], [43], [44]. Transgenic wild-type and *chip* plants expressing GFP-ATG8a were exposed to 45°C for 0, 1.5 and 3 hours, recovered for 0.5 hour at room temperature and then observed by confocal fluorescence microscopy for autophagosomes. In both the wild-type and *chip* mutant plants, the numbers of punctate GFP signals were low before heat stress (Figure 10). The punctate fluorescent structures in wild-type plants did not increase significantly during the first 1.5 hours of heat stress but elevated by 6-fold after 3-hour heat stress (Figure 10). In the *chip* mutant plants, the punctate fluorescent structures increased by almost 4-fold during the first 1.5 hours of heat stress (Figure 10). By the third hour of heat stress, however, there were similar numbers of punctate fluorescent structures in the wild-type and *chip* mutant plants (Figure 10). Thus, deficiency of *CHIP* caused an earlier induction of autophagosome accumulation by heat stress.

We also compared wild type and *chip* mutant plants for expression patterns of seven *Arabidopsis* autophagy genes (*ATG5*, *ATG6*, *ATG7*, *ATG8a*, *ATG9*, *ATG10*, *ATG18a*) and *NBR1* in response to high temperature. *Arabidopsis* wild-type and *chip* mutant plants were placed in a 45°C chamber and total RNA was isolated from rosette leaves for detection of *ATG* and *NBR1* gene transcripts using qRT-PCR. At 45°C, the transcript levels of the *ATG* genes were elevated with varying kinetics (Figure S2). For most of the *ATG* genes, the increased levels of transcripts were detected as early as 2 hours after initiation of the heat stress (Figure S2). *ATG8a* exhibited increased transcript levels after 2-hour exposure to the high temperature (Figure 10). In the *chip* mutant, we observed increased levels of transcripts for *ATG6*, *ATG7*, *ATG9* similar to those in the wild-type plants (Figure S2). For the other five genes tested, however, changes in their transcripts in the *chip* mutant responded more sensitively to heat stress than those in the wild-type plants (Figure S2). For example, transcripts for *ATG5*, *ATG8a*, *ATG10* and *NBR1* in the *chip* mutant increased more rapidly and to higher levels than those in the wild-type plants during the first 4–6 hours of heat stress (Figure S2). During the later stage of heat stress, decline in the transcript levels for some of the *ATG* and *NBR1* genes after initial increase in the *chip* mutant also occurred earlier and to greater extents than in the wild-type plants (Figure S2). *ATG18a* was an exception because its transcripts declined earlier in the chip mutant than in the wild-type plants after an initial induction by heat stress (Figure S2).

We also compared wild type and *chip* mutant plants for changes in the transcript levels for *CAT2* and *CAT3* under heat stress. For the wild-type plants, the transcript levels for *CAT2* were little changed during the first 6 hour heat stress but declined for the remaining four hours to about 50% of its control levels (Figure S3). The transcript levels for *CAT2* in the *chip* mutant remained unchanged during the first 2 hours but declined rapidly during the remaining 8-hour heat stress to about 10% of its control levels. For *CAT3*, heat stress increased transcript levels in both wild-type and *chip* mutant plants but this increase was more pronounced in the wild-type plants than in the *chip* mutant (Figure S3).

## Discussion

### A critical role of the CHIP E3 ubiquitin ligase in plant stress tolerance

The animal CHIP ubiquitin E3 ligase has been extensively analyzed for its critical role in protein quality control. CHIP deficiency in knockout mice reduces median survival from 25 months to 10 months, representing a 60% decrease in longevity [30], [31]. Decreased longevity in the knockout mice is associated with accelerated aging-related pathophysiological phenotypes including reduced body weight, increased and accelerated skeletal muscle atrophy, decreased body fat stores, increased signs of cardiac hypertrophy, osteoporosis and kyphosis [30], [31]. The *Arabidopsis* CHIP has also been characterized for its expression, E3 ligase activity and interaction with the Hspc70 chaperones and potential ubiquitination substrates [32], . However, functional analysis of *Arabidopsis CHIP* has been exclusively through overexpression, which, surprisingly, caused cold- and heat-hypersensitivity in transgenic plants [32], [33]. In the present study, we have isolated two independent T-DNA knockout mutants for *Arabidopsis* CHIP and conducted a comprehensive genetic analysis of their phenotypes under both normal and stress conditions. Contrary to the strong phenotypes of knockout mice, *Arabidopsis chip* and *chip nbr1* mutants display no detectable alternation in growth or development throughout the life cycle. A large number of studies have shown that misfolded or damaged proteins are prevalent even under normal growth conditions with roughly 30% of all newly synthesized proteins degraded by the UPS and related pathways [45], [46]. In *Arabidopsis*, mutant deficient for autophagy or other ubiquitin E3 ligases display premature senescence under normal growth conditions [47], [48], [49]. Therefore, there are likely other ubiquitin E3 ligases and autophagy adaptors/receptors that act independently or redundantly with CHIP and NBR1 for basal protein quality control in plants.

*Arabidopsis chip* mutants are also normal to both the hemibiotrophic bacterial pathogen *P. syringae* and necrotrophic pathogen *B. cinerea*, suggesting that it is also dispensable in plant immunity. On the other hand, the *chip* mutants are hypersensitive to heat, oxidative and salt stresses (Figures 1 & 2). Increased heat sensitivity of the *chip* mutant plants was associated with increased accumulation of insoluble protein aggregates (Figure 3). Therefore, CHIP is important for removal of stress-damaged proteins during plant responses to abiotic stresses. CHIP functions as both a co-chaperone and an E3 ubiquitin ligase, thereby linking cellular protein folding with protein degradation. Through physical interactions with molecular chaperones Hsp70/Hsc70 proteins, CHIP can ubiquitinate those chaperone-bound nonnative proteins and target them for degradation by UPS or selective autophagy. Deficiency of CHIP in the *chip* mutants would lead to accumulation of denatured or damaged proteins and cytotoxicity under stress conditions. Intriguingly, transgenic *CHIP*-overexpressing plants are hypersensitive to both cold and high temperatures [32], most likely because of the deleterious effect to cellular heath due to a number of possible mechanisms. First, molecular chaperones associated with CHIP is also involved in folding and refolding of nonnative proteins that may form under stress conditions. Excess levels of CHIP could interfere with molecular chaperones for their protein folding or refolding activities or prematurely ubiquitinate those chaperone-bound but refoldable proteins and targets them for unnecessary degradation. Second, CHIP may interact with other proteins and regulate their degradation independent of chaperones. In *Arabidopsis*, a number of proteins including subunits of protein phosphatase 2A, chloroplast FtsH and ClpP4 proteases interact directly with and act as substrates of the CHIP ubiquitin E3 ligase [34], [35], [36]. Chronic overexpression of CHIP could affect important signaling pathways involving PP2A and alter protein degradation and other important functions of chloroplasts, leading to deleterious effects on plant health [34], [35], [36]. These results indicate that a delicate balance in cellular CHIP levels is important for protein quality control and for plant stress tolerance.

### CHIP- and NBR1-mediated anti-proteotoxic pathways in plant stress responses

Despite the highly similar phenotypes of the *chip* and *nbr1* mutants, additional analysis strongly suggests that CHIP and NBR1 mediate two distinct but complementary anti-proteotoxic pathways. First, compromised tolerance of the *chip nbr1* double mutant to heat stresses was consistently more severe than those of the *chip* and *nbr1* single mutants (Figure 4). The additive nature of the *chip nbr1* double mutant phenotypes indicated that the roles of CHIP and NBR1 in plant stress tolerance do not completely overlap. Second, insoluble protein aggregates accumulated in the *chip* single and *chip nbr1* double mutant plants were still highly ubiquitinated under heat stress (Figures 3 & 5), thereby implicating a CHIP-independent mechanism for ubiquitination of stress-induced protein aggregates in NBR1-mediated selective autophagy. Third, proteomic profiling revealed that after 6-hour heat stress, aggregates for a substantial number of proteins differentially accumulated in the *nbr1* and *chip* mutants (Figure 7A; Tables S1 & S2). The levels of protein aggregates in the *nbr1* mutant for a substantial number of proteins including Rubisco actvase and catalases were 3–5 times higher than in the *chip* mutant after 6-hr heat stress (Figure 7A). Other proteins including a group of light-harvesting complex subunits, on the other hand, preferentially accumulates as aggregates in the *chip* mutant after the same period of heat stress (Figure 7A). A previously reported study has shown that CHIP and HSC70-4 specifically target degradation of the plastid-destined light-harvesting protein subunit proteins in a plastid import mutant [50].

**Figure 11:**
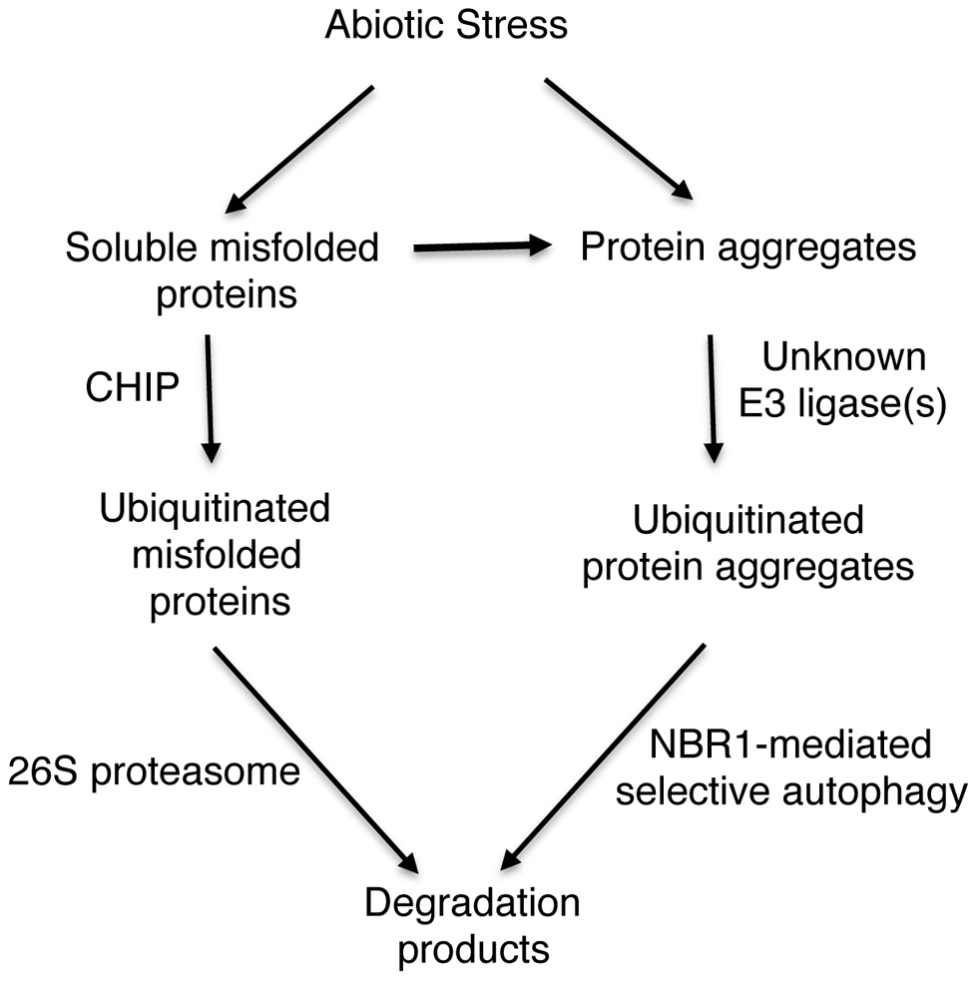
A proposed model for CHIP- and NBR1-mediated antiproteotoxic pathways. Stress conditions such as high temperature cause generation of misfolded and damaged proteins. Chaperone-associated E3 ubiquitin ligase CHIP ubiquitinates soluble misfolded proteins that are associated with chaperone molecules and targets their degradation by the 26S proteasomes. Misfolded proteins or protein aggregates are recognized by an unknown E3 ubiquitin ligase and targeted for degradation by NBR1-mediated selective autophagy. Those soluble misfolded proteins that CHIP-mediated 26S proteasomes fail to degrade due to limited capacity will also aggregate and become targets of NBR1-mediated selective autophagy.

Rubisco activase and catalases are known to be highly heat sensitive and prone to form aggregates [51], [52], [53]. After 6-hour heat stress, the two proteins preferentially accumulated as protein aggregates in the *nbr1* mutants but not in the *chip* or *rpn1a* proteasome mutant (Figure 7A; Figure 8). This finding suggests a critical factor for selection of protein substrates by the two pathways: those highly aggregate-prone proteins such as Rubisco activase and catalases are efficiently cleared by selective autophagy only most likely because these protein aggregates/oligomers are difficult to be dissociated and unfolded to pass through the small 13 Å wide central cavity of the barrel-shaped 20S proteolytic core [54] (Figure 11). Soluble misfolded proteins such as the cytosolic precursors of LHC proteins can apparently be unfolded and, therefore, can be efficiently degraded by CHIP-mediated UPS [50] (Figure 11). After extended heat stress, differential accumulation of protein aggregates was reduced even though the total levels of protein aggregates increased (Figure 5A). This result is consistent with a complementary nature of the two pathways in clearing misfolded proteins (Figure 11). When there is an extended heat stress and, consequently, increased levels of misfolded proteins, soluble misfolded proteins normally targeted by CHIP-mediated UPS can increase and form aggregate due to limited capacity of UPS and accumulated if selective autophagy is blocked as in the *nbr1* mutant (Figure 11). Likewise, aggregate-prone proteins such as Rubisco activase and catalases normally targeted by NBR-mediated selective autophagy can also increase in the *chip* mutant after extended heat stress because CHIP deficiency leads to accumulation and aggregation of soluble misfolded proteins at increased levels, which could overwhelm the capacity of NBR1-mediated selective autophagy. Consistent with the complementary nature of the two pathways, the levels of aggregates for a majority of detected proteins were higher in the *chip nbr1* double mutant than in the *chip* and *nbr1* single mutants (Figures S1 & S2).

Using the well-established GFP-ATG8 system, we showed that *chip* mutant plants became more sensitive to heat stress for induced autophagosome formation (Figure 10). Furthermore, changes in the transcript levels for some of the autophagy genes in the *chip* mutant plants occurred earlier than in wild-type plants in response to heat stress (Figure S2). Thus, CHIP deficiency led to quicker induction of autophagy in stressed plants for degradation of accumulated toxic protein species. These results indicated that CHIP- and NBR1-mediated pathways are not only complementary in function but also coordinated in regulation. Similarly, impairment of the UPS has been found to induce autophagy *in vitro* in non-plant organisms. When cultured human cells are challenged with excess misfolded proteins that overwhelms the UPS or treated with proteasome inhibitors, induction of autophagy is observed as evidenced by redistribution of ATG8/LC3 into punctate structures and accumulation of autophagosomes [55], [56]. Similar induction of autophagy is also observed in response to genetic impairment of the 26S proteasome in *Drosophila* [57]. How the UPS and autophagy are coordinated in both function and regulation is little understood. Some studies have suggested that misfolded proteins, if accumulated, can be actively transformed into a cytoplasmic, juxtanuclear structure called aggresomes [58]. Several proteins including histone-deacetylase 6 (HDAC6), P62, and Alfy (autophagy-linked FYVE) implicated in aggresome formation and clearance have also emerged as potential players in mediating the crosstalk between the UPS and autophagy [58]. In *Arabidopsis*, compromised heat tolerance due to CHIP or NBR1 deficiency is associated with increased accumulation of insoluble ubiquitinated protein aggregates (Figures 3 & 5). Identifying components important for the formation, detection and ubiquitination of stress-induced protein aggregates will provide valuable insights into the functional and regulatory coordination between the UPS and selective autophagy in plant stress responses.

### Stress-induced protein aggregates and proteotoxicity in plants

Protein aggregates have been extensively studied in animal systems because of their roles in a wide variety of diseases called amyloidosis including Alzheimer's, Parkinsons's and prion disease [58]. By contrast, there has been no reported effort to identify systematically stress-denatured or damaged proteins and protein aggregates in plants. From proteomic profiling, we have identified a number of proteins that were highly accumulated as insoluble protein aggregates in heat-stressed *chip* and *nbr1* mutants (Tables S1 & S2). One of these abundant protein aggregates is Rubisco activase, which activates Rubisco by facilitating the ATP-dependent removal of sugar phosphates from Rubisco active sites. Rubisco activase is known to be highly heat-sensitive and aggregate-prone with a temperature optimum for ATP hydrolysis of 44°C compared to >60°C for carboxylation by Rubisco [53]. The finding that Rubisco activase is among the most abundant aggregated proteins in heat-stressed plants supported its highly aggregate-prone nature and its critical role for the sensitivity of photosynthesis to inhibition by heat [53]. Rubsco activase may have additional biological functions including protecting the thylakoid associated protein synthesis machinery against heat inactivation and repressing leaf senescence [59]. *Arabidopsis* knockout mutant seedlings for Rubisco activase were yellow, severely stunted and unable to set seeds, supporting its crucial role in plant growth [59]. Proteomic profiling also revealed that CAT3 and CAT2, the major catalase isoforms in photosynthetic *Arabidopsis* tissues [38], accumulated abundantly as insoluble protein aggregates in heat-stressed *chip nbr1* mutants (Tables S1 & S2). Western blotting confirmed that catalases were preferentially accumulated as insoluble, inactive proteins in the *chip nbr1* mutants after 6- and 9-hour heat stress (Figure 9). Reduction in soluble, active forms of Rubisco activase and catalases would lead to inhibited photosynthesis and increased oxidative stress, which are known to occur in heat-stressed plants [60], [61].

Proteotoxicity has been generally attributed to non-specific interactions of nonnative proteins with functional proteins and cellular structures such as membranes [1]. Monitoring the changes of the transcripts, proteins and activity of *Arabidopsis* CAT2 and CAT3 revealed a possible regulatory circuit at multiple levels that ultimately leads to large reduction of cellular catalase activity under heat stress. Western blotting revealed that CHIP and NBR1 deficiency in the *chip nbr1* mutant increased the levels of total and insoluble catalase proteins but reduced the levels of soluble catalase proteins after heat stress (Figure 9). Reduced levels of soluble, active catalases in heat-stressed *chip nbr1* mutant plants indicated that CHIP- and NBR-mediated pathways impact not only the levels of misfolded and aggregated protein targets but also the levels of their native counterparts. This effect on soluble native catalases could be due to direct physical interactions with accumulated misfolded proteins, thereby promoting catalase protein aggregation and inactivation. In addition, we observed that the levels of *CAT2* and *CAT3* transcripts in the *chip* mutant plants were substantially lower than those in the wild-type plants even during the relatively early stages of heat stress (Figure S3). Therefore, reduced levels of soluble, active catalases could also be due to reduced expression of the *CAT* genes caused by compromised protein degradation in heat-stressed *chip* mutant plants. In light of these findings, proteotoxicity due to compromised protein quality control can be mediated not only by direct physical interactions with cellular molecules but also by indirect effects on important cellular processes such as gene expression.

A substantial number of proteins identified from aggregated proteins are involved in protein synthesis, folding and maturation (Tables S1 & S2). The highly heat-sensitive and aggregation-prone nature of some of these proteins such as translation factors could underlie the detrimental effects of heat stress on plant growth and development. Aggregation of some of these proteins may also act as adaptive mechanisms for reprograming transcription and protein synthesis in response to abiotic stresses. Other proteins such as heat shock proteins function as molecular chaperones that may be involved in folding or refolding of soluble misfolded proteins and became associated with aggregated proteins when refolding is unsuccessful. Other proteins with activities in protein folding and maturation may be associated with insoluble proteins for facilitation of protein aggregation. During severe and prolonged stress conditions, a large number of denatured and damaged proteins are expected to be generated and can overwhelm UPS due to limited capacity of ubiquitination and degradation, leading to a highly proteotoxic environment in stressed cells. Active promotion of aggregation of denatured and damaged proteins may reduce proteotoxicity, particularly if the protein aggregates are sequestered subcellularly. From western blotting of catalases, only a small fraction of the high levels of aggregated catalases accumulated in heat-stressed *chip nbr1* mutant was ubiquitinated (Figure 9A). It appears that unlike in UPS, ubiquitination of only a fraction of proteins in a protein aggregate is sufficient for targeted degradation by NBR1-mediated selective autophagy. As ubiquitination is ATP-dependent, targeted degradation of aggregated proteins by selective autophagy is, therefore, more energetically efficient than UPS and this advantage could be potentially important for plants under stress conditions, which often inhibit photosynthesis and promote respiration.

## Materials and Methods

### *Arabidopsis* genotypes and growth conditions

The *Arabidopsis* mutants and wild-type plants used in the study are all in the Col-0 background. The *nbr1* and *rpn1a* mutants have been previously described [39], [62]. Homozygous *chip-1* (Salk_048371), *chip-2* (Salk_059253), *cat2-1* (Salk_076998) and *cat3-1* (GABI_110C11) mutants were identified by PCR using primers flanking the T-DNA insertions listed in Table S3. The *chip nbr1* double mutant was generated through a genetic cross between the *chip-1* and *nbr1-1* single mutants. *Arabidopsis* plants were grown in growth chambers at 22°C, 120 µE m^−2^ light on a photoperiod of 12-hour light and 12 h dark.

### RNA isolation and quantitative RT–PCR

Total plant RNA isolation and reverse transcription were performed as previously described [20]. qRT-PCR was performed using the iCycler iQTM real-time PCR detection system (Bio-Rad, Hercules, CA, USA) and the relative gene expression was calculated as previously described [55]. The *Arabidopsis ACTIN2* gene was used as internal control. Gene-specific primers for qRT-PCR are listed in Table S4.

### Analysis of abiotic stress tolerance

For testing heat tolerance, *Arabidopsis* Col-0 wild type and mutant plants were placed in 22°C and 45°C growth for 9 hours and then moved to room temperature for 3–5 day recovery for observation of heat stress symptoms or survival rates. For testing tolerance to oxidative stress, six weeks-old *Arabidopsis* plants were sprayed with 20 µM methyl viologen (MV) and kept under light for two days before the picture of representative plants was taken. For testing ABA sensitivity or salt tolerance, sterilized *Arabidopsis* seeds were sown on solid ½× MS medium or on the ½× MS medium containing 0.5 µM ABA or 150 mM NaCl. Germination rates were determined by scoring green cotyledons for the following 8 days.

### Separation of soluble and insoluble proteins and western blotting

*Arabidopsis* leaves were collected before and after heat treatment, ground in liquid nitrogen and homogenized in a detergent containing extraction buffer (100 mMTris/HCl, pH 8.0, 10 mM NaCl, 1 mM EDTA, 1% Triton X-100, 0.2% ß-mercaptoethanol). Soluble and detergent-resistant insoluble proteins were separated through low-speed centrifugation as previously described [20]. Protein fractionation by SDS-PAGE and western blotting for detection of ubiquitinated proteins and catalases were performed as previously described [20]. Ubiquitinated proteins were detected by protein blotting using an anti-ubiquitin monoclonal antibody (Sigma, USA). RCA was detected with a previously generated monoclonal antibody [40]. Catalases were detected using an anti-catalase monoclonal antibody (3B6) that was raised against tobacco catalases [51], [63]. The antigen-antibody complexes were detected by enhanced chemiluminescence using luminal as substrate as previously described [20].

### Visualization of induction of autophagy using GFP-ATG8a

Transgenic Col-0 wild-type and *atg7* plants expressing a GFP–ATG8a fusion construct were previously described [20]. To generate transgenic *chip* mutant plants expressing GFP–ATG8a, the fusion construct was transformed into the *chip-1* mutant using the floral-dip method and transgenic plants were identified on the basis of kanamycin resistance and confirmed by RNA blotting using the GFP DNA fragment as a probe. For visualization of induction of autophagy, 5-weeks old transgenic plants expressing the GFP-ATG8a fusion gene were treated with or without heat shock for various amounts of time and recovered for 0.5 hour. The leaves of transgenic plants were observed using LSM710 confocal microscope with excitation at 488 nm, and images were superimposed using ZEISS LSM710 software.

### Mass spectrometric analysis of insoluble proteins

Insoluble proteins were isolated and separated by SDS-PAGE. Destained gels were dried in a vacuum centrifuge and the in-gel proteins were reduced with 10 mM dithiothreitol (DTT) in 100 mM NH_4_HCO_3_ for 30 min at 56°C and then alkylated with 200 mM iodoacetamide in 100 mM NH_4_HCO_3_ in the dark at room temperature for 30 minutes. In-gel proteins were digested overnight in 12.5 ng/µl trypsin in 25 mM NH_4_HCO_3_. The peptides were extracted three times with 60% acetonitrile (ACN)/0.1% trifluoroacetic acid (TFA), pooled and dried by a vacuum centrifuge. LTQ Velos (Thermo Scientific) equipped with a micro-spray interface was connected for eluted peptides detection. Data-dependent MS/MS spectra were obtained simultaneously. Each scan cycle consisted of one full scan mass spectrum (*m/z* 300–1800) followed by 20 MS/MS events of the most intense ions with the following dynamic exclusion settings: repeat count 2, repeat duration 30 seconds, exclusion duration 90 seconds. MS/MS spectra were automatically searched against the Unprot ARATH database (53847 sequences, March 8th, 2013) using the BioworksBrowser rev. 3.1(Thermo Electron, San Jose, CA.). Protein identification results were extracted from SEQUEST outfiles with BuildSummary. The peptides were constrained to be tryptic and up to two missed cleavages were allowed. Carbamidomethylation of cysteines were treated as a fixed modification, whereas oxidation of methionine residues was considered as variable modifications. The mass tolerance allowed for the precursor and fragment ions were 2.0 and 0.8 Da, respectively. The protein identification criteria were based on Delta CN (≥0.1) and cross-correlation scores (Xcorr, one charge≥1.9, two charges ≥2.2, three charges ≥3.75).

### Assays of catalase activity

Total and soluble proteins from *Arabidopsis* leaves were prepared as previously described [20] and used for the determination of the catalase activity. All of the steps were performed at 4°C. An aliquot of the extract was used to determine the protein content, following the method as previously described [64]. Catalase activity was measured as a decline in *A*_240_ using the method as previously described [65]. The spectrophotometric assays were conducted using a SHIMADZU UV-2410PC spectrophotometer (Shimadzu Co., Kyoto, Japan).

### Accession numbers

Sequence data for the genes described in this study can be found in the GenBank/EMBL data libraries under the accession numbers shown in parentheses: CHIP (At3g07370), ACTIN2 (AT3G18780), ATG5 (At5g17290), ATG6 (At3g61710), ATG7 (At5g45900), ATG8a (AT4G21980), ATG9 (At2g31260), ATG10 (At3g07525), ATG18a (At3g62770), NBR1 (AT4G24690), CAT2 (At4g35090), CAT3(At1g20620), RPN1a (At2g20580).

## Supporting Information

Figure S1 Recognition of *Arabidopsis* CAT2 and CAT3 by monoclonal antibody 3B6. Total soluble proteins were isolated from *Arabidopsis* wild type (WT), *cat2-1* and *cat3-1* mutants, fractionated by SDS-PAGE and probed with anti-catalase monoclonal antibody 3B6. CAT2 and CAT3, which differ slightly in migration, were indicated.
(PDF)

Figure S2 Induction of autophagy genes by heat stress. Five weeks-old *Arabidopsis* wild-type (WT) and *chip-1* mutant plants were placed in a 45°C growth chambers and total RNA was isolated from leaf samples collected at indicated times. Transcript levels were determined using qRT-PCR. Error bars indicate SE (n = 3).
(PDF)

Figure S3 Effects of CHIP deficiency on *CAT2* and *CAT3* expression under heat stress. Five weeks-old *Arabidopsis* wild-type (WT) and *chip-1* mutant plants were placed in a 45°C growth chambers and total RNA was isolated from leaf samples collected at indicated times. Transcript levels were determined using qRT-PCR. Error bars indicate SE (n = 3).
(PDF)

Table S1 Insoluble proteins accumulated after 6-hour heat stress.
(PDF)

Table S2 Insoluble proteins accumulated after 9-hour heat stress.
(PDF)

Table S3 Primers for identifying the T-DNA insertions.
(PDF)

Table S4 Primers for qRT-PCR.
(PDF)
