# Correction: E3 Ubiquitin Ligase CHIP and NBR1-Mediated Selective Autophagy Protect Additively against Proteotoxicity in Plant Stress Responses

**DOI:** 10.1371/journal.pgen.1004478

**Published:** 2014-06-06

**Authors:** The PLOS Genetics Staff

Figure 1 is incorrect. Panel E contained incorrect images of methyl viologen-treated plants as heat-treated plants. The corrected version is provided here. This error does not affect the interpretation of the results or conclusions of the paper. The authors apologise for any confusion this may have caused. The legend is correct and has not changed.

**Figure 1:**
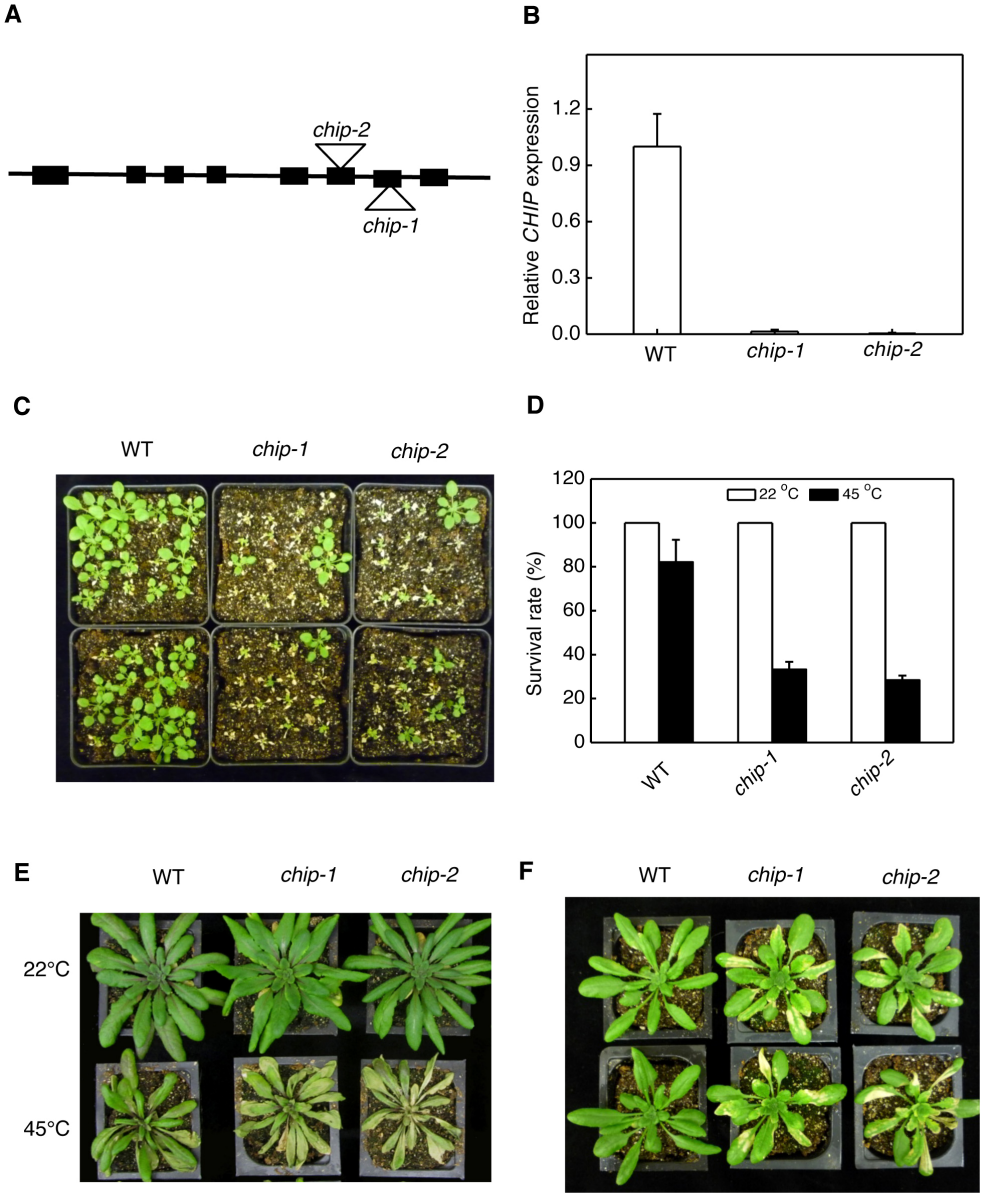
Identification and analysis of *chip* mutants for heat and oxidative stress tolerance. (A) Diagram of the *CHIP* gene and insertion sites. (B) Transcript levels of CHIP in Col-0 wild type (WT) and chip mutants as determined using qRT-PCR. Error bars indicate SE (n  =  3). (C) Assays of heat tolerance of young seedlings. Approximately 70 two-weeks old seedlings were placed in a 45°C growth chamber for 9 hours. The heat-treated plants were then moved to a 22°C growth chamber for recovery. The picture was taken at three days after the heat treatment. (D) Survival rates of heat-stressed young seedlings. Approximately 70 two-weeks seedlings were placed in a 45°C growth chamber for 9 hours. The heat-treated plants were then moved to a 22°C growth chamber for recovery. The survival rates were determined at three days after the heat treatment. Error bars indicate SE (n  =  3). (E) Assays of heat tolerance of mature plants. Six-weeks old mature plants were placed in a 45°C growth chamber for 9 hours. The heat-treated plants were then moved to a 22°C growth chamber for recovery. The picture was taken at three days after the heat treatment. (F) Assays of tolerance to oxidative stress. Six-weeks old mature plants were sprayed with 20 µM methyl viologen (MV) and kept under light for two days before the picture of representative plants was taken. The experiments were repeated three times with similar results.
