# Supplementary figures and images for: E3 Ubiquitin Ligase CHIP and NBR1-Mediated Selective Autophagy Protect Additively against Proteotoxicity in Plant Stress Responses
Source: PLoS Genet. 2014 Jan 30;10(1):e1004116. doi: 10.1371/journal.pgen.1004116 (PMC3907298; doi:10.1371/journal.pgen.1004116)

Figure S1

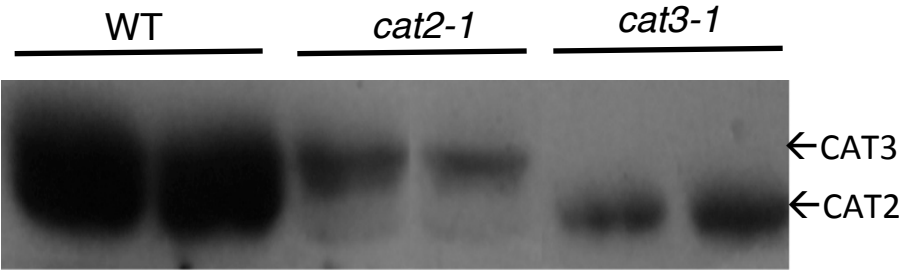

Supplement: Figure S1 — Recognition of Arabidopsis CAT2 and CAT3 by monoclonal antibody 3B6. Total soluble proteins were isolated from Arabidopsis wild type (WT), cat2-1 and cat3-1 mutants, fractionated by SDS-PAGE and probed with anti-catalase monoclonal antibody 3B6. CAT2 and CAT3, which differ slightly in migration, were indicated. (PDF) [file pgen.1004116.s001.pdf]

Figure S2

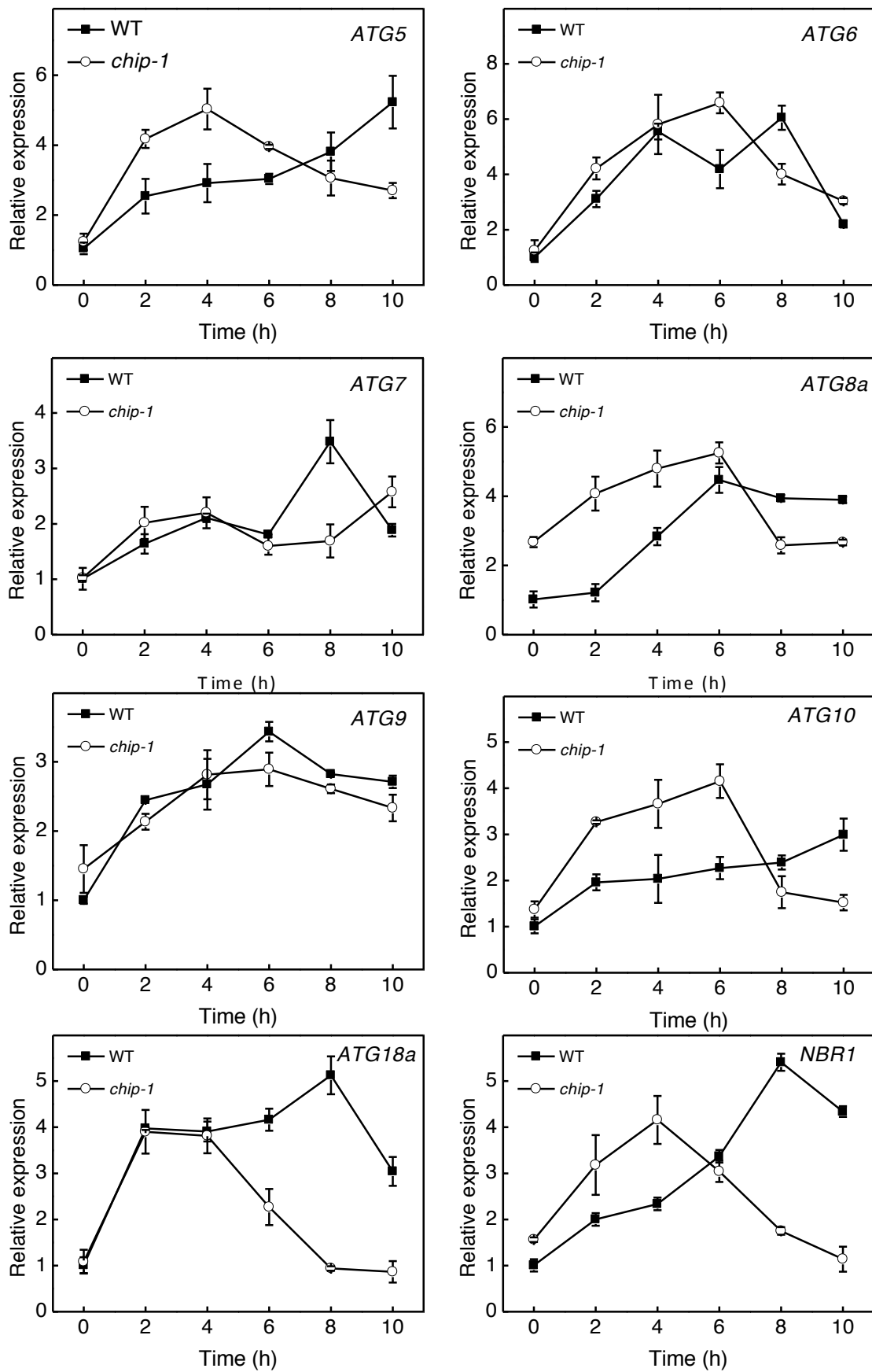

Supplement: Figure S2 — Induction of autophagy genes by heat stress. Five weeks-old Arabidopsis wild-type (WT) and chip-1 mutant plants were placed in a 45°C growth chambers and total RNA was isolated from leaf samples collected at indicated times. Transcript levels were determined using qRT-PCR. Error bars indicate SE (n = 3). (PDF) [file pgen.1004116.s002.pdf]

Figure S3

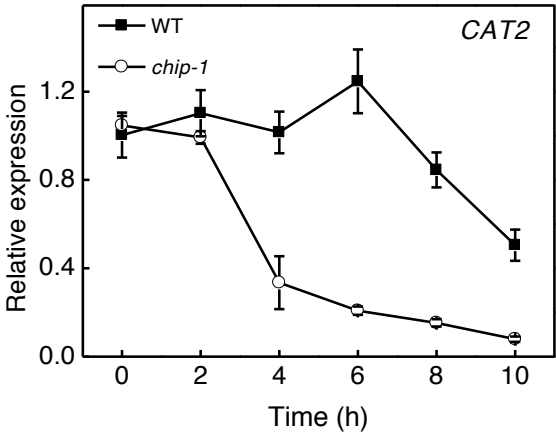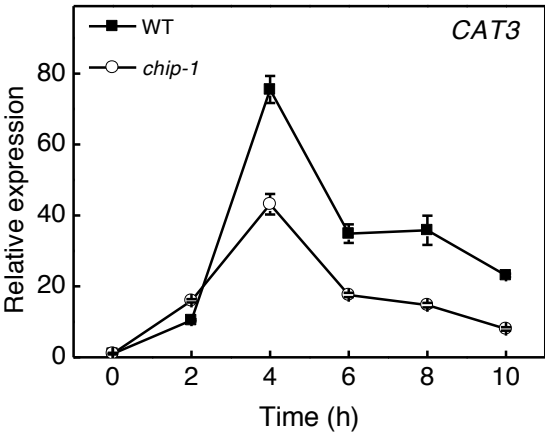

Supplement: Figure S3 — Effects of CHIP deficiency on CAT2 and CAT3 expression under heat stress. Five weeks-old Arabidopsis wild-type (WT) and chip-1 mutant plants were placed in a 45°C growth chambers and total RNA was isolated from leaf samples collected at indicated times. Transcript levels were determined using qRT-PCR. Error bars indicate SE (n = 3). (PDF) [file pgen.1004116.s003.pdf]
