# Supplementary material for: E3 Ubiquitin Ligase CHIP and NBR1-Mediated Selective Autophagy Protect Additively against Proteotoxicity in Plant Stress Responses
Source: PLoS Genet. 2014 Jan 30;10(1):e1004116. doi: 10.1371/journal.pgen.1004116 (PMC3907298; doi:10.1371/journal.pgen.1004116)
Supplement: Table S1 — Insoluble proteins accumulated after 6-hour heat stress. (PDF) [file pgen.1004116.s004.pdf]

Table S1 Insoluble proteins accumulated after 6-hour heat stress

GI	Description	Normalized peptide number*			
		WT	*nbr1*	*chip*	*nbr1/chip*
AT1G29910	Light harvesting chlorophyll A/B binding protein 1, LHCB1	212	302	797	865
AT2G34420	Photosystem II light harvesting complex gene B1B2, LHB1B2	170	276	542	849
AT2G05070	Light-harvesting chlorophyll B-binding 2, LHCB2	124	346	421	573
AT2G39730	Rubisco activase, RCA	61	387	81	548
AT5G01530	Light harvesting chlorophyll A/B binding protein II, LHCB4	100	134	282	354
AT1G20620	Catalase-3, CAT3	50	270	70	290
AT4G35090	Catalase-2, CAT2	27	150	33	230
AT3G62030	Rotamase CYP 4, ROC4	35	128	108	188
AT4G10340	Light harvesting complex of photosystem II 5, LHCB5	38	85	80	124
AT4G20360	RAB GTPase homolog E1B, RABE1B	18	119	57	125
ATCG00490	Large subunit of Rubisco, LBCL	9	107	48	105
AT5G54270	Light-harvesting chlorophyll B-binding protein 3, LHCB3	24	54	60	99
ATCG00480	ATP synthase subunit beta, ATPB	30	98	64	80
AT3G45140	Lipoxygenase 2, LOX2	7	52	39	79
AT3G47470	Light-harvesting chlorophyll-protein protein complex I subunit A4, LHCA4	4	39	59	67
AT1G09340	Chloroplast RNA binding, CRB	32	38	37	45
AT5G12030	Heat shock protein 17.6A, HSP17.6A	8	49	35	59
AT4G27440	Protochlorophyllide oxidoreductase B, PORB	13	36	33	67
ATCG00280	Photosystem II reaction center protein C, PSBC	19	31	39	53
AT3G01500	Salicylic acid-binding protein 3, SABP3	9	48	24	59
AT1G15820	Light harvesting complex photosystem II subunit 6, LHCB6	6	29	43	60
ATCG00120	ATP synthase subunit alpha, ATPA	5	38	25	61
AT1G20020	Ferredoxin-NADP(+)-oxidoreductase 2, FNR2,	6	25	36	59
AT1G62750	Snowy cotyledon 1, SCO1	9	43	33	38
AT5G66190	Ferredoxin-NADP(+)-oxidoreductase 1, FNR1	6	34	33	49
AT5G14740	Beta carbonic anhydrase 2, Beta CA2	10	36	26	46
AT1G42970	Glyceraldehyde-3-phosphate dehydrogenase B subunit, GAPB	2	41	33	41
AT1G12900	Glyceraldehyde 3-phosphate dehydrogenase a subunit 2, GAPA-2	3	37	22	51
AT1G11860	Glycine cleavage T-protein family	8	28	21	54
AT3G26650	Glyceraldehyde 3-phosphate dehydrogenase A subunit, GAPA	6	38	19	48
AT3G14415	Glycolate oxidase 2, GOX2	0	33	29	46
AT3G46230	Heat shock protein 17.4, HSP17.4	6	30	31	41

AT1G61520	Photosystem I light harvesting complex gene 3, LHCA3	6	18	36	45
AT4G04640	ATP synthase gamma chain 1, ATPC1	7	30	24	38
AT5G38420	Rubisco small subunit 2B, RBCS2B	5	30	26	36
AT3G47520	Malate dehydrogenase, MDH	4	33	27	32
AT1G67090	Ribulose bisphosphate carboxylase small chain 1A, RBCS1A	7	25	30	32
AT2G21330	Fructose-bisphosphate aldolase 1, FBA1	5	23	22	43
AT3G60750	Transketolase;	1	32	19	41
AT5G38430	Rubisco small subunit 1B, RBCS1B	4	24	26	39
AT3G14420	Glycolate oxidase 1, GOX1	3	29	19	40
AT4G02770	Photosystem I subunit D-1, PSAD-1	5	21	32	29
AT3G16470	Jacalin-related lecyin 35, JAL35	4	23	19	40
AT5G60390	GTP binding elongation factor Tu family protein	4	25	21	28
AT4G09650	ATP synthase delta-subunit gene, ATPD	3	13	21	38
AT5G02500	Heat shock protein 70-1, HSP70-1	3	25	21	22
AT3G13920	Eukaryotic translation initiation factor 4A1, EIF4A1	12	12	18	27
ATCG00680	Photosystem II reaction center protein B, PSBB	5	18	28	18
AT1G16030	Heat shock protein 70B, HSP70B	6	16	16	28
AT1G31330	Photosystem I subunit F, PSAF	6	21	23	16
AT1G44575	Photosystem II subunit S, PSBS	2	14	17	33
AT3G56940	Copper response defect1, CRD1	3	21	14	28
AT1G32900	Granule bound starch synthase 1, GBSS1	3	23	14	24
AT4G25200	Mitochondrion-localized small heat shock protein 23.6, HSP23.6-MITO	3	13	24	24
AT5G08670	Mitochondrial ATP synthase beta-subunit	5	22	11	26
AT5g17920	Cobalamin-independent methionine synthase, CIMS	7	21	15	20
AT5G35630	Glutamine synthetase 2, GLN2	5	20	10	27
AT1G09640	Translation elongation factor EF1B gamma chain	13	15	14	18
AT1G23310	Glutamate:glyoxylate aminotransferase 1, GGT1	2	18	12	28
AT3G54890	Photosystem I light harvesting complex gene 1, LHCA1	3	9	21	26
AT3G09440	Heat shock protein 70 (Hsp 70) family protein	3	22	12	22
AT5G09660	Peroxisomal NAD-malate dehydrogenase 2, PMDH2	4	13	21	19
ATCG00270	Photosystem II reaction center protein D, PSBD	3	16	19	19
AT4G38970	Fructose-bisphosphate aldolase 2, FBA2	0	25	12	19
AT3G12780	Phosphoglycerate kinase 1, PGK1	2	21	8	24

AT3G16640	Translationally controlled tumor protein, TCTP	4	16	11	24
AT1G10760	Starch excess 1	0	21	5	28
AT1G68010	Hydroxypyruvate reductase, HPR	5	19	13	16
AT3G08580	ADP/ATP carrier 1, AAC1	3	21	14	14
AT3G61470	Photosystem I light harvesting complex gene 2, LHCA2	4	16	15	16
AT3G12580	Heat shock protein 70, HSP70	4	14	10	22
AT2G37660	NAD(P)-binding Rossmann-fold superfamily protein	3	18	8	20
AT3G14210	Epithiospecifier modifier 1, ESM1	3	17	12	17
AT1G57720	Translation elongation factor EF1B, gamma chain	2	11	15	20
AT3G08740	Elongation factor P (EF-P) family protein	4	14	11	19
AT4G13940	S-adenosyl-L-homocystein hydrolase 1, SAH1	1	17	5	23
AT1G07890	Ascorbate peroxidase 1, APX1	8	6	13	18
AT5G67030	Zeaxanthin expoxidase, ZEP	6	11	12	16
AT5G02490	H70 family protein	6	15	9	14
AT1G52400	Beta-glucosidase homolog 1, BGL1	2	5	26	10
AT2G15970	Cold regulated 413 plasma membrane 1, COR413-PM1	2	9	18	14
AT3g55800	Sedoheptulose-bisphosphatase, SBPASE	2	13	10	17
AT5G42650	Allene oxide synthase, AOS	4	10	4	23
AT1G54270	Member of eIF4A - eukaryotic initiation factor 4A	1	14	6	19
AT1G08380	PHOTOSYSTEM I SUBUNIT O, PSAO	6	13	8	12
AT1G67700	Hypersensitive to high light 1, HHL1	5	17	8	9
AT1G56050	GTP-binding protein-related	1	9	12	16
AT5G12250	Beta-6 tubulin, TUB6	6	6	10	16
AT1G03630	Photochlorophyllide oxidoreductase C, PORC	4	11	9	13
AT3G18780	Actin 2, ACT2	3	13	6	14
AT1G30580	GTP binding	1	12	5	16
AT4G02520	Glutathion S-transferase phi 2, GST2	3	9	9	13
ATCG01110	NAD(P)H dehydrogenase subunit H, NDHH	1	8	10	15
AT1G01080	Protochlorophyllide oxidoreductase C, PORC	2	8	9	14
AT4G12720	Nudix hydrolase homolog 7, NUDT7	9	7	6	11
AT1G33120	Ribosomal protein L6 family	6	4	8	14
AT3G26070	Plastid-lipid associated protein PAP / fibrillin family protein	3	9	6	14
AT4G14960	Tubulin alpha-6, TUA6	2	6	9	15
AT3G46780	Plastid transcriptionally active 16, PTAC16	3	13	8	7
AT3G52930	Fructose-bisphosphate aldolase 8, FBA8	3	10	6	12

AT5G19940	Plastid-lipid associated protein PAP / fibrillin family protein	0	9	12	10
AT1G73060	Low PSII accumulation 3, LPA3	1	11	5	13
AT2G13360	Alanine: glyoxylate aminotransferase, AGT	2	11	3	14
AT5G03720	Heat shock transcription factor A3, HSFA3	0	7	10	13
ATCG00020	Photosystem II reaction center protein A, PSBA	2	8	12	8
ATCG00650	Ribosomal protein S18, RPS18	0	4	11	15
AT1G73110	P-loop containing nucleoside triphosphate hydrolases superfamily protein	3	10	11	5
AT2G37220	Encodes a chloroplast RNA binding protein	0	8	10	11
AT4G34870	Rotamase cyclophilin 5, ROC5	2	5	7	15
AT4G28750	PSA E1 knockout, PSAE-1	2	13	7	6
AT5G64050	Ovule abortion 3, OVA3	3	8	5	12
AT3G04120	Glyceraldehyde-3-phosphate dehydrogenase C subunit, CGPC	4	8	6	8
AT3G11940	Arabidopsis minute-like 1, AML1	1	4	8	13
AT3G52660	RNA-binding (RRM/RBD/RNP motifs) family protein	3	9	4	10
AT3G59970	Methylenetetrahydrofolate reductase 1, MTHFR1	1	10	5	10
AT5G52640	Heat shock protein 90.1, HSP 90.1	4	5	8	9
AT1G05010	Ethylene forming enzyme, EFE	2	4	10	9
AT4G37930	Serine hydroxymethyl transferase 1, SHM1	1	9	6	9
AT1G66580	Ribosomal protein L10 C, RPL10C	6	4	7	7
AT1G56410	Heat shock protein 70T-1, HSP70T-1	0	3	9	11
AT1G62780	Unknown protein;	3	9	4	7
AT1G74470	Encodes for a multifunctional protein with geranylgeranyl reductase activity	2	9	3	9
AT4G13430	Isopropyl malate isomerase large subunit 1, IIL1	0	9	1	13
AT4G27670	Heat shock protein 21, HSP21	1	7	7	8
ATCG00800	Encodes a chloroplast ribosomal protein S3	4	2	12	15
AT3G62530	ARM repeat superfamily protein	2	5	6	9
AT5G19770	Tubulin alpha-3, TUA3	1	7	8	6
AT1G07660	Histone superfamily protein	3	4	6	8
AT1G59359	Ribosomal protein S5 family protein	4	5	6	6
AT2G21170	Triosephosphate isomerase, TIM	1	7	3	10
AT5G13650	Suppressor of variegation 3, SVR3	1	5	3	12
AT5G44340	Tubulin beta chain 4, TUB4	4	4	6	7
AT2G15620	Nitrite reductase 1, NIR1	2	5	6	7
AT4G23670	Polyketide cyclase/dehydrase and lipid transport superfamily protein	2	6	5	7

AT4G34090	Unknown protein	0	1	12	7
AT5G07350	Tudor-sn protein 1, TUDOR1	3	6	5	6
AT5G50920	Heat shock protein 93-V, HSP93-V	2	7	1	10
AT5G56030	Heat shock protein 90.2, HSP 90.2	1	4	3	12
AT3G17390	Methionine adenosyltransferase 4, MAT4	0	5	7	7
AT5G35170	Adenylate kinase family protein	1	7	4	7
AT1G03475	Lesion initiation 2, LIN2	4	5	6	3
AT2G06050	Delayed dehiscence 1, DDE1	2	6	5	5
AT2G30950	Variegated 2, VAR2	2	6	4	6
AT4G14210	Phytoene desaturase, PDS	2	3	4	9
AT4G17090	Beta-amylase 3, BAM3	1	4	5	8
AT4G18480	Chlorina 42, CH42	2	6	2	8
AT1G02930	Glutathion S-transferase 1, GST1	2	3	4	8
AT1G67430	Ribosomal protein L22p/L17e family protein	4	4	5	4
AT5G35530	Ribosomal protein S3 family protein	0	3	7	7
AT5G42020	Luminal binding protein	0	5	5	7
AT5G62390	BCL-2-associated athanogene 7, BAG7	0	4	5	8
AT5G66120	3-Dehydroquinate synthase	0	4	5	8
AT1G06000	Encodes a flavonol-7-O-rhamnosyltransferase	5	4	5	2
AT1G48600	Phosphoethanolamine N-methyltransferase, PMEAMT	1	6	4	5
AT1G56070	Low expression of osmotically responsive genes 1, LOS1	3	6	3	4
AT1G70820	Phosphoglucomutase	2	4	3	7
AT2G39800	Delta1-pyrroline-5-carboxylate synthase 1, P5CS1	1	6	4	5
AT2G41100	Calmodulin LIKE 4, CAL4	4	2	6	4
AT2G47730	Glutathione S-transferase phi 8, GSTF8,	0	5	4	7
AT3G22890	ATP sulfurylase 1, APS1,	3	4	8	1
AT5G08690	Encodes the mitochondrial ATP synthase beta-subunit	0	8	4	4
AT5G12110	Glutathione S-transferase	3	4	5	4
ATCG00350	Encodes psaA protein comprising the reaction center for photosystem	0	4	3	9
ATCG00790	Ribosomal protein L16, RPL16	1	3	5	7
AT1G66430	Pfk B-like carbohydrate kinase family protein	2	7	1	5
AT2G30790	Photosystem II subunit P-2, PSBP-2	0	7	3	5
AT3G11130	Clathrin heavy chain 1, CHC1	2	3	3	7
AT3G24320	Chloroplast mutator, CHM	2	4	4	5
AT5G45170	Haloacid dehalogenase-like hydrolase (HAD) superfamily protein	0	4	6	5

AT5G55220	Trigger factor type chaperone family protein	1	6	4	4
ATCG00750	Ribosomal protein S11, RPS11	1	4	5	5
AT1G52510	Alpha/beta-Hydrolases superfamily protein	1	3	2	8
AT1G74310	Heat shock protein 101, HSP101	2	6	3	3
AT1G78570	Rhamnose biosynthesis 1, RHM1	4	1	2	7
AT2G01350	Quinolinate phoshoribosyl transferase, QPT	4	3	2	5
AT2G35410	RNA-binding (RRM/RBD/RNP motifs) family protein	0	4	5	5
AT4G33010	Glycine decarboxylase P-protein 1, GLDP1	2	4	2	6
AT4G37000	Accelerated cell death 2, ACD2	2	5	2	5
AT5G25980	Beta glucosidase 37, BGLU37	2	4	2	6
AT5G38660	Acclimation of photosynthesis to environment, APE1	0	3	3	8
AT2G01250	Ribosomal protein L30/L7 family protein	2	4	1	6
AT3G22520	Unknown protein	1	3	2	7
AT5G08650	Small GTP-binding protein	0	4	3	6
AT5G20290	Ribosomal protein S8e family protein	0	4	2	7
AT1G01560	MAP kinase 11, MPK11	2	2	4	4
AT1G27450	Adenine phosphoribosyl transferase 1, APT1	0	4	4	4
AT1G30230	Elongation factor 1B beta, EF1BB,	3	0	2	7
AT1G30380	Photosystem O subunit K, PSAK	2	2	7	1
AT1G32060	Photosphoribulokinase, PRK	0	8	1	3
AT2G40590	Ribosomal protein S26e family protein	1	3	4	4
AT3G09680	Ribosomal protein S12/S23 family protein	1	3	4	4
AT3G19170	Presequence protease 1, PREP1	0	5	0	7
AT4G30530	Gamma-glutamyl peptidase 1, GGP1	0	2	4	6
AT5G26742	Embryo defective 1138, EMB1138	1	5	3	3
AT1G04410	Cytosolic-NAD-dependent malate dehydrogenase 1, C-NAD-MDH1	0	2	2	7
AT2G45790	Phosphomannomutase, PMM	0	0	6	5
AT3G04790	Embryo defective 3119, EMB3119	2	3	3	3
AT3G08590	2,3-Biphosphoglycerate-independent phosphoglycerate mutase 2, IPGAM2	2	4	2	3
AT4G35250	High chlorophyll fluorescence phenotype 244, HCF244	0	4	3	4
AT5G58710	Rotamase CYP 7, ROC7	0	1	2	8
AT5G61970	Signal recognition particle-related / SRP-related	3	1	3	4
ATCG00340	Encodes the D1 subunit of photosystem I reaction center.	2	5	2	2
AT1G09490	Similar to Eucalyptus gunnii alcohol dehydrogenase	5	1	2	2
AT1G54050	HSP20-like chaperones superfamily protein	4	1	4	6

AT1G76030	V-ATPase B subunit 1, VAB1	1	5	1	3
AT2G23600	Acetone-cyanohydrin lyase, ACL	1	1	3	5
AT2G43090	Aconitase/3-isopropylmalate dehydratase protein	0	4	1	5
AT2G44120	Ribosomal protein L30/L7 family protein	3	0	2	5
AT3G07770	Heat shock protein 90.6, HSP90.6	0	5	3	2
AT3G19010	2-Oxoglutarate (2OG) and Fe(II)-dependent oxygenase superfamily protein	3	0	3	4
AT4G22710	Cytochrome P450	0	4	0	6
AT5G18660	Pale-green and chlorophyll B reducted 2, PCB2	0	2	3	5
AT5G55280	Homolog of bacterial cytokinesis Z-ring protein FTSZ 1-1, FTSZ1-1,	2	2	2	4
AT5G58250	Embryo defective 3143, EMB3143	0	4	2	4
AT5G64040	Encodes the only subunit of photosystem I	3	0	4	3
ATMG01190	ATP synthase subunit 1, ATP1	0	5	1	4
AT1G08520	Pigment defective embry 166, PDE166	1	2	4	2
AT1G11430	Multiple organellar RNA editing factor 9, MORF9	0	4	1	4
AT1G24020	MLP-like protein 423, MLP423	1	0	3	5
AT1G29630	Exonuclease family protein	2	1	5	1
AT2G20260	Photosystem I subunit E-2, PSAE-2	0	6	0	3
AT2G25080	Glutathion peroxidase 1, GPX1	0	4	1	4
AT2G36620	Ribosomal protein L24, RPL24A	0	1	6	2
AT2G45990	Unknown protein	0	4	0	5
AT4G05050	Ubiquitin 11, UBQ11	2	0	1	6
AT4G13670	Plastid transcriptionally active 5, PTAC5	2	1	4	2
AT4G26530	Fructose-bisphosphate aldolase 5, FBA5	1	4	1	3
ATMG00510	NADH dehydrogenase subunit 7, NAD7	1	2	2	4
AT1G19570	Dehydroascorbate reductase 1, DHAR1	0	6	0	2
AT1G20010	Tubulin beta-5 chain, TUB5	1	0	0	7
AT1G23130	Polyketide cyclase/dehydrase and lipid transport superfamily protein	3	0	2	3
AT1G23190	PGM3, PHOSPHOGLUCOMUTASE 3	0	2	2	4
AT1G32220	NAD(P)-binding rossmann-fold superfamily protein	1	2	3	2
AT1G52040	Myrosinase-binding protein 1, MBP1	0	1	3	4
AT1G54010	GDSL-like lipase/acylhydrolase superfamily protein	6	0	2	0
AT1G55450	S-adenosyl-L-methionine-dependent methyltransferases superfamily protein	2	1	0	5
AT1G72640	NAD(P)-binding Rossmann-fold superfamily protein	0	4	1	3
AT2G20610	Aberrant lateral root formation 1, ALF1	0	0	2	6

AT2G31670	Stress responsive alpha-beta barrel domain protein	0	3	1	4
AT2G42590	General regulatory factor 9, GRF9	1	0	0	7
AT2G47390	Prolyl oligopeptidase family protein	0	3	2	3
AT3G09720	P-loop containing nucleoside triphosphate hydrolases superfamily protein	1	1	2	4
AT3G63140	Chloroplast stem-loop binding protein of 41 KDA, CSP41A	0	2	1	5
AT5G22880	Histone B2, H2B	0	1	4	3
AT5G39980	Tetratricopeptide repeat (TPR)-like superfamily protein	1	3	0	4
AT5G44720	Molybdenum cofactor sulfurase family protein	0	1	2	5
AT5G60600	Chloroplast biogenesis 4, CLB4,	0	3	0	5
AT1G16880	Act doman repeats 11, ACR11	1	2	0	4
AT1G18060	Unknown protein	0	4	2	1
AT1G22940	Thiamine requiring 1, TH1	1	0	5	1
AT1G27970	Nuclear transport factor 2B, NTF2B	1	2	3	1
AT2G45960	Named plasma membrane intrinsic protein 1;2, PIP1;2	2	1	1	3
AT3G45950	Pre-mRNA splicing Prp18-interacting factor	2	1	2	2
AT4G02930	GTP binding Elongation factor Tu family protein	1	3	0	3
AT5G02610	Ribosomal L29 family protein	2	0	2	3
ATCG00160	Ribosomal protein S2, RPS2	2	1	2	2
ATMG00210	Ribosomal protein L5, RPL5	1	0	1	5
AT1G35720	Annexin 1	0	2	1	3
AT1G51060	Histone H2A 10, HTA10	2	2	1	1
AT1G63940	Monodehydroascorbate reductase 6, MDAR6	1	3	0	2
AT2G16600	Rotamase CYP 3, ROC3	0	0	0	6
AT2G36530	Low expression of osmoticially responsive genes 2, LOS2	0	2	1	3
AT4G05180	Photosystem II subunit Q, PSBQ	0	0	1	5
AT4G09010	Ascorbate peroxidase 4, APX4	0	4	0	2
AT4G39520	Encodes a member of the DRG (developmentally regulated G-protein) family.	4	0	2	0
AT5G18380	Ribosomal protein S5 domain 2-like superfamily protein	0	1	1	4
AT5G23010	Methylthioalkylmalate synthase 1, MAM1	2	1	2	1
AT5G51820	Phosphoglucomutase, PGM	1	2	0	3
AT1G12800	Nucleic acid-binding, OB-fold-like protein	2	0	3	0
AT1G64880	Ribosomal protein S5 family protein	1	0	2	2
AT1G65350	Ubiquitin 13, UBQ13	0	1	2	2
AT2G36460	Fructose-bisphosphate aldolase 6, FBA6,	0	0	5	0
AT3G03250	UDP-glucose pyrophosphorylase 1, UGP1	0	2	2	1

AT3G04550	Unknown protein	2	0	3	0
AT3G11510	Ribosomal protein S11 family protein	2	0	0	3
AT3G42050	Vacuolar ATP synthase subunit H family protein	0	0	1	4
AT3G44860	Farnesoic acid carboxyl-O-methyltranseferase, FAMT	2	0	2	1
AT3G45780	Root phototropism 1, RPT1	0	0	1	4
AT3G55440	Triosephosphate isomerase, TPI	0	0	2	3
AT3G59400	Genomes uncoupled 4, GUN4	3	0	1	1
AT4G27090	Ribosomal protein L14	0	1	2	2
AT5G15200	Ribosomal protein S4	0	1	0	4
AT5G19510	Translation elongation factor EF1B/ribosomal protein S6 family protein	0	0	0	5
AT5G42310	Pentatricopeptide repeat (PPR-like) superfamily protein	0	0	0	5
AT2G41740	VLN2	0	1	0	3
AT3G15000	Multiple organellar RNA editing factor 8, MORF8	0	2	0	2
AT3G19710	Branched-chain aminotransferase 4, BCAT4,	2	0	1	1
AT4G24770	31-KDA RNA binding protein, RBP31	2	0	0	2
AT5G04800	Ribosomal S17 family protein	0	0	2	2
AT5G28840	"GDP-D-MANNOSE 3',5'-EPIMERASE", GME	3	0	1	0
AT5G48575	Protein of unknown function (DUF1216)	0	2	1	1
AT1G02150	Tetratricopeptide repeat (TPR)-like superfamily protein	2	0	0	1
AT1G18080	Receptor for activated C kinase 1 A, RACK1A	0	1	0	2
AT1G53240	Mitochondrial malate dehydrogenase 1, MMDH1	0	3	0	0
AT1G72150	Patellin 1, PATL1	0	0	0	3
AT2G09990	Ribosomal protein S5 domain 2-like superfamily protein	0	1	0	2
AT2G15410	Gypsy-like retrotransposon family	1	0	0	2
AT2G17150	Plant regulator RWP-RK family protein	0	1	0	2
AT2G46240	Bcl-2-associated athanogene 6, BAG6	2	0	1	0
AT3G20820	Leucine-rich repeat (LRR) family protein	1	0	0	2
AT3G44620	Protein tyrosine phosphatases	0	1	0	2
AT3G57260	Pathogenesis-related protein 2, PR2	0	0	1	2
AT3G59780	Rhodanese/Cell cycle control phosphatase superfamily protein	0	1	0	2
AT4G13010	Oxidoreductase, zinc-binding dehydrogenase family protein	0	0	0	3
AT4G31500	Altered tryptophan regulation 4, ATR4	0	1	0	2
AT4G39260	GLYCINE-RICH RNA-BINDING PROTEIN 8, GR-RBP8	0	1	0	2
AT5G12020	17.6 KDA clasee II heat shock protein, HSP17.6II	0	0	1	2
AT5G20630	Germin-like protein 3, GLP3	3	0	0	0

AT5G60790	General control non-repressible 1, GCN1	0	1	0	2
ATCG00580	Photosystem II reaction center protein E, PSBE	1	0	0	2
AT1G09310	Protein of unknown function	0	0	2	0
AT1G16800	P-loop containing nucleoside triphosphate hydrolases superfamily protein	0	0	0	2
AT1G22780	Pointed first leaves 1, PFL1	0	0	0	2
AT1G59910	Actin-binding FH2 (formin homology 2) family protein	0	0	0	2
AT1G74710	Salicylic acid induction deficient 2, SID2	0	0	0	2
AT2G14560	Late upregulated in response to hyaloperonospora parasitica, LURP1	0	0	2	0
AT2G24020	Uncharacterised BCR	0	0	0	2
AT2G27680	NAD(P)-linked oxidoreductase superfamily protein	0	0	0	2
AT2G30200	Embryo defective 3147, EMB3147	0	0	0	2
AT3G03110	Exportin 1B, XPO1B	0	0	0	2
AT3G52380	Chloroplast RNA-binding protein 33, CP33	0	0	0	2
AT3G53460	Chloroplast RNA-binding protein 29, CP29	0	0	0	2
AT4G14880	Onset of leaf death 3, OLD3	0	0	0	2
AT4G34150	Calcium-dependent lipid-binding (CaLB domain) family protein	2	0	0	0
AT4G34450	Coatomer gamma-2 subunit	0	2	0	0
AT4G34670	Ribosomal protein S3Ae	0	0	0	2
AT4G34920	PLC-like phosphodiesterases superfamily protein	0	0	0	2
AT4G39970	Haloacid dehalogenase-like hydrolase (HAD) superfamily protein	0	0	0	2
AT5G06970	Contains Interpro domains	0	0	0	2
AT5G09810	Member of Actin gene family	0	0	0	2
AT5G10450	G-BOX regulating factor 6, GRF6	0	0	0	2
AT5G58770	Cis-prenyltransferase 4, CPT4	0	0	0	2

*Normalized peptide number is the peptide number detected by shortgun MS/MS from the amount of insoluble proteins isolated from 0.1 mg total protein for each genotype. Among the ~170 most abundant proteins detected, those with ≥2 fold enrichment in the *chip* mutant are highlighted in bright green, while those with ≥2 fold enrichment in the *nbr1* mutant are highlighted in yellow
