# Supplementary material for: E3 Ubiquitin Ligase CHIP and NBR1-Mediated Selective Autophagy Protect Additively against Proteotoxicity in Plant Stress Responses
Source: PLoS Genet. 2014 Jan 30;10(1):e1004116. doi: 10.1371/journal.pgen.1004116 (PMC3907298; doi:10.1371/journal.pgen.1004116)
Supplement: Table S2 — Insoluble proteins accumulated after 9-hour heat stress. (PDF) [file pgen.1004116.s005.pdf]

Table S2 Insoluble proteins accumulated after 9-hour heat stress

GI	Description	Normalized peptide number*			
		WT	*nbr1*	*chip*	*nbr1/chip*
AT2G39730	Rubisco activase, RCA	65	464	407	649
ATCG00490	Large subunit of Rubisco, LBCL	60	542	232	626
AT3G62030	Rotamase CYP 4, ROC4	38	154	184	226
AT1G20620	Catalase-3, CAT3	39	140	147	165
AT3G45140	Lipoxygenase 2, LOX2	37	137	106	148
AT4G20360	RAB GTPase homolog E1B, RABE1B	27	107	118	146
AT4G10340	Light harvesting complex of photosystem II 5, LHCB5	20	66	72	143
AT4G02520	Glutathione S-transferase phi 2, GST2	30	109	58	181
AT3G01500	Salicylic acid-binding protein 3, SABP3	25	70	44	67
AT4G35090	Catalase-2, CAT2	10	41	39	59
AT1G67090	Ribulose bisphosphate carboxylase small chain 1A, RBCS1A	11	47	29	58
AT1G23310	Glutamate:glyoxylate aminotransferase 1, GGT1	12	41	44	45
AT5G01530	Light harvesting chlorophyll A/B binding protein II, LHCB4	13	31	46	52
AT5G52640	Heat shock protein 90.1, HSP 90.1	6	39	36	49
AT2G34420	Photosystem II light harvesting complex gene B1B2, LHB1B2	9	15	50	54
AT3G14415	Glycolate oxidase 2, GOX2	16	41	25	40
AT5G56030	Heat shock protein 90.2, HSP 90.2	11	39	32	40
AT2G05070	Light-harvesting chlorophyll B-binding 2, LHCB2	9	17	42	52
AT3G14420	Glycolate oxidase 1, GOX1	22	35	27	35
AT1G42970	Glyceraldehyde-3-phosphate dehydrogenase B subunit, GAPB	3	35	30	49
AT3G11130	Clathrin heavy chain 1, CHC1	11	37	27	42
AT4G36650	Plant-specific TFIIb-related protein (PBRP)	10	29	25	42
AT3G08940	Light harvesting complex photosystem II, LHCB4.2	3	22	44	45
AT5G38410	Rubisco small subunit 3B, RBCS3B	4	28	25	32
AT1G11860	Glycine cleavage T-protein family	8	26	28	24
AT1G29910	Light harvesting chlorophyll A/B binding protein 1, LHCB1	6	26	22	32
AT3G08530	Clathrin heavy chain 2, CHC2	5	27	19	35
AT5G08670	Encodes the mitochondrial ATP synthase beta-subunit.	9	29	21	27
AT3G13920	Eukaryotic translation initiation factor 4A1, EIF4A1	8	26	20	30
ATCG00270	Photosystem II reaction center protein D, PSBD	7	19	28	30
AT2G37660	NAD(P)-binding Rossmann-fold superfamily protein	7	17	27	32

AT3G26650	Glyceraldehyde 3-phosphate dehydrogenase A subunit, GAPA	10	21	20	31
AT3G60750	Transketolase;	8	22	23	29
AT1G62750	Snowy cotyledon 1, SCO1	9	25	17	28
AT3G09440	Heat shock protein 70 family protein	8	22	18	27
ATCG00480	ATP synthase subunit beta, ATPB	10	18	21	23
AT1G12900	Glyceraldehyde 3-phosphate dehydrogenase a subunit 2, GAPA-2	0	29	14	28
AT1G07890	Ascorbate peroxidase 1, APX1	14	25	11	20
AT1G09340	Heteroglycan-interacting protein 1.3, HIP1.3	14	22	10	23
ATCG00120	ATP synthase subunit alpha, ATPA	7	20	17	25
AT4G27670	Heat shock protein 21, HSP21	6	23	16	23
AT5G14740	Beta carbonic anhydrase 2, Beta CA2	2	15	24	25
AT1G15820	Light harvesting complex photosystem II subunit 6, LHCB6	9	15	18	19
AT5G25980	Beta glucosidase 37, BGLU37	1	16	20	22
AT2G05710	Aconitase 3, ACO3	5	20	9	23
AT3G47520	Malate dehydrogenase, MDH	5	13	20	18
AT4G37930	Serine hydroxymethyl transferase 1, SHM1	5	17	14	20
AT1G09640	Translation elongation factor EF1B gamma chain	6	10	15	22
AT1G32060	Photosphoribulokinase, PRK	3	15	12	23
AT5G60390	GTP binding Elongation factor Tu family protein	5	13	13	22
AT1G74310	Heat shock protein 101, HSP101	1	21	6	24
AT3G12780	Phosphoglycerate kinase 1, PGK1	0	19	13	20
AT3G52880	Monodehydroascorbate reductase 1, MDAR1	0	19	8	24
AT4G33010	Glycine decarboxylase P-protein 1, GLDP1	5	16	14	16
AT1G07400	HSP20-like chaperones superfamily protein	5	13	13	19
AT1G02930	Glutathione S-transferase 1, GST1	10	14	9	16
AT1G20020	Ferredoxin-NADP(+)-oxidoreductase 2, FNR2,	5	9	18	17
AT1G73060	Low PSII accumulation 3, LPA3	4	8	15	22
AT5G09660	Peroxisomal NAD-malate dehydrogenase 2, PMDH2	6	13	12	17
AT5G38660	Acclimation of photosynthesis to environment, APE1	4	13	20	11
AT3G08580	ADP/ATP carrier 1, AAC1	2	14	11	20
AT2G13360	Alanine: glyoxylate aminotransferase, AGT	3	14	10	18
AT5G35630	Glutamine synthetase 2, GLN2	2	17	10	16
AT1G68010	Hydroxypyruvate reductase, HPR	4	15	11	14
AT2G04400	Aldolase-type TIM barrel family protein	9	8	14	13
AT4G25200	Mitochondrion-localized small heat shock protein 23.6,	4	9	16	15

	HSP23.6-MITO				
AT3G19170	Presequence protease 1, PREP1	4	8	12	19
AT3G47470	Light-harvesting chlorophyll-protein protein complex I subunit A4, LHCA4	5	11	14	13
AT4G26970	Aconitase 2, ACO2	5	14	8	15
AT1G31330	Photosystem I subunit F, PSAF	7	5	13	16
AT4G05050	Ubiquitin 11, UBQ11	3	12	12	13
AT1G16030	Heat shock protein 70B, HSP70B	2	13	14	10
AT5g17920	Cobalamin-independent methionine synthase, CIMS	4	13	9	13
AT5G42650	Allene oxide synthase, AOS	2	10	14	12
AT4G04640	ATPC1, ATP synthase gamma chain 1	7	7	10	13
AT2G29500	HSP20-like chaperones superfamily protein	10	2	9	15
AT4G13430	Isopropyl malate isomerase large subunit 1, IIL1	6	4	14	12
ATCG00680	Photosystem II reaction center protein B, PSBB	3	11	12	10
AT1G07920	GTP binding Elongation factor Tu family protein	2	8	9	16
AT1G10760	Starch excess 1	5	5	10	15
AT3G46230	Heat shock protein 17.4, HSP17.4	5	10	8	12
AT5G67030	Zeaxanthin expoxidase, ZEP	2	6	13	14
ATCG00020	Photosystem II reaction center protein A, PSBA	5	8	12	8
AT2G06050	Delayed dehiscence 1, DDE1	4	8	8	12
AT1G57720	Translation elongation factor EF1B, gamma chain	4	9	13	5
AT1G56070	Low expression of osmotically responsive genes 1, LOS1	1	16	9	4
AT4G23850	Long-chain acyl-coa synthetase 4, LACS4	2	8	8	12
AT1G52400	Beta-glucosidase homolog 1, BGL1	3	5	5	15
AT1G59870	ATP-binding cassette G36, ABCG36	0	11	5	12
AT4G12720	Nudix hydrolase homolog 7, NUDT7	2	6	7	13
AT1G64190	6-Phosphogluconate dehydrogenase family prot	3	5	7	12
AT4G27440	Protochlorophyllide oxidoreductase B, PORB	2	9	5	11
AT5G02490	Heat shock protein 70 family protein	3	4	9	11
AT4G17090	Beta-amylase 3, BAM3	3	4	8	11
AT1G61520	Photosystem I light harvesting complex gene 3, LHCA3	2	9	9	5
AT4G35830	Aconitase 1, ACO1	4	4	7	10
AT1G52510	Alpha/beta-Hydrolases superfamily protein	6	9	6	3
AT5G35170	Adenylate kinase family	2	9	5	7
ATCG00340	Encodes the D1 subunit of photosystem I reaction center, PSAB	0	4	5	14
AT2G29720	CTF2B	6	3	5	8

AT3G18780	Actin 2, ACT2	3	5	5	9
AT4G34450	Coatomer gamma-2 subunit	0	10	2	10
AT5G66190	Ferredoxin-NADP(+)-oxidoreductase 1, FNR1	2	4	7	9
AT2G47390	Prolyl oligopeptidase family protein	2	6	4	9
AT3G61470	Photosystem I light harvesting complex gene 2, LHCA2	5	6	5	5
AT4G19170	Nine-cis-epoxycarotenoid dioxygenase 4, NCED4	2	6	4	9
AT4G28750	PSA E1 Knockout, PSAE-1	0	5	6	10
AT1G78900	Vacuolar ATP synthase subunit A, VHA-A	3	3	8	6
AT2G21170	Triosephosphate isomerase, TIM	2	5	5	8
AT2G37220	Encodes a chloroplast RNA binding protein	2	6	4	8
AT1G56410	Heat shock protein 70T-1, HSP70T-1	5	0	0	14
AT4G23670	Polyketide cyclase/dehydrase and lipid transport superfamily protein	2	5	4	8
AT3G54640	Tryptophan-requiring 3, TRP3	2	4	5	7
AT3G56940	Copper response defect1, CRD1	5	2	8	3
AT4G14210	Phytoene desaturase, PDS	2	5	4	7
AT5G13030	Unknown protein;	3	4	6	5
AT5G55220	Trigger factor type chaperone family protein	0	3	4	11
AT1G03130	Photosystem I subunit D-2, PSAD-2	0	5	4	8
AT1G03475	Lesion initiation 2, LIN2	3	2	10	2
AT1G09430	ATP-citrate lyase A-3, ACLA-3	0	3	7	7
AT1G79550	Phosphoglycerate kinase, PGK	0	6	3	8
AT2G30950	Variegated 2, VAR2	3	3	2	9
AT2G33150	Peroxisome defective 1, PED1	0	3	8	6
AT2G36460	Fructose-bisphosphate aldolase 6, FBA6,	3	4	4	6
AT3G04840	Ribosomal protein S3Ae	0	7	5	5
AT4G24190	Heat shock protein 90.7, HSP90.7,	3	4	6	4
AT1G20510	OPC-8:0 coaligase1, OPCL1	8	0	6	2
AT2G16600	Rotamase CYP 3, ROC3	0	5	4	7
AT3G12580	Heat shock protein 70, HSP70	2	16	5	20
AT5G07350	Tudor-sn protein 1, TUDOR1	2	4	4	6
AT1G01080	RNA-binding (RRM/RBD/RNP motifs) family protein	4	6	3	10
AT1G20010	Tubulin beta-5 chain, TUB5	0	3	0	12
AT1G59860	HSP20-like chaperones superfamily protein	4	0	7	4
AT5G14740	Carbonic anhydrase 2, CA2	0	15	0	0
AT5G62390	BCL-2-associated athanogene 7, BAG7	2	7	0	6

AT1G52560	HSP20-like chaperones superfamily protein	3	0	11	0
AT1G63940	Monodehydroascorbate reductase 6, MDAR6	2	4	0	8
AT2G36250	Encodes one of two FtsZ proteins, FTSZ2-1	0	2	5	7
AT3G07770	Heat shock protein 89.1, HSP89.1	2	2	6	4
AT3G54890	Photosystem I light harvesting complex gene 1, LHCA1	5	2	5	2
AT4G34090	Unknown protein	5	2	3	4
AT5G57850	4-Amino-4-deoxychorismate lyase, ADCL	2	4	5	3
AT5G59720	Heat shock protein 18.2, HSP18.2	0	3	5	6
AT2G21330	Fructose-bisphosphate aldolase 1, FBA1	0	6	2	5
AT3G59020	ARM repeat superfamily protein;	2	3	5	3
AT3G59970	Methylenetetrahydrofolate reductase 1, MTHFR1	4	5	2	2
AT3G63140	Chloroplast stem-loop binding protein of 41 KDA, CSP41A	5	4	4	0
AT4G30530	Gamma-glutamyl peptidase 1, GGP1	2	3	3	5
AT4G38970	Fructose-bisphosphate aldolase 2, FBA2	0	13	0	0
AT5G66120	3-Dehydroquinate synthase	3	3	0	7
AT2G25080	Glutathion peroxidase 1, GPX1	1	4	3	4
AT3G18070	Beta glucosidase 43, BGLU43	9	0	3	0
AT4G24620	Phosphoglucose isomerase 1, PGI1	5	0	3	4
AT4G30910	Cytosol aminopeptidase family protein	0	6	0	6
AT1G22300	14-3-3 Protein G-BOX factor 14 epsilon, 14-3-3EPSILON	0	4	3	4
AT3G22200	Gamma-aminobutyrate transaminase, GABA-T	2	4	3	2
AT3G42050	Vacuolar ATP synthase subunit H family protein	0	6	5	0
AT3G52500	Eukaryotic aspartyl protease family protein	3	0	8	0
AT4G00570	NAD-dependent malic enzyme 2, NAD-ME2	5	4	2	0
AT5G25980	Glucoside glucohydrolase 2, TGG2	0	0	1	10
AT5G26742	Embryo defective 1138, EMB1138	0	2	3	6
AT1G63770	Peptidase M1 family protein	4	3	0	3
AT2G39800	Delta1-pyrroline-5-carboxylate synthase 1, P5CS1	2	3	3	2
AT3G26070	Plastid-lipid associated protein PAP / fibrillin family protein	3	0	5	2
AT4G23100	Glutamate-cysteine ligase, GSH1	3	2	5	0
AT4G29010	Abnormal inflorescence meristem, AIM1	2	2	3	3
AT5G25980	Beta glucosidase 37, BGLU37	10	0	0	0
AT5G59970	Histone superfamily protein	0	10	0	0
AT1G09340	Chloroplast RNA binding, CRB	0	4	2	3
AT2G20610	Aberrant lateral root formation 1, ALF1	2	4	3	0

AT2G42590	General regulatory factor 9, GRF9	0	5	0	4
AT2G46520	Cellular apoptosis susceptibility protein	2	2	2	3
AT3G08740	Elongation factor P (EF-P) family protein	3	0	4	2
AT3G14205	Phosphoinositide phosphatase family protein	0	0	9	0
AT3G59920	Rab GDP dissociation inhibitor 2, GDI2	3	4	2	0
AT4G02930	GTP binding Elongation factor Tu family protein	0	5	2	2
AT5G20290	Ribosomal protein S8e family protein	0	0	9	0
AT1G18500	Methylthioalkylmalate synthase-like 4, MAML-4	4	4	0	0
AT2G04030	Heat shock protein 90.5, HSP90.5	0	3	3	2
AT2G23600	Acetone-cyanohydrin lyase, ACL	0	2	4	2
AT2G47730	Glutathione S-transferase phi 8, GSTF8,	0	3	2	3
AT3G18780	Deformed root hairs 1, DER1,	0	0	3	5
AT3G52750	Nuclear gene that encodes a plastidial division protein, FtsZ2-2	3	0	5	0
AT4G37000	Accelerated cell death 2, ACD2	0	3	2	3
AT5G19550	Aspartate aminotransferase 2, ASP2	0	3	2	3
AT5G19940	Plastid-lipid associated protein PAP / fibrillin family protein	3	3	2	0
AT5G36880	Acetyl-coa synthetase, ACS	8	0	0	0
AT5G59880	Actin depolymerizing factor 3, ADF3	0	3	2	3
AT1G03630	Photochlorophyllide oxidoreductase C, PORC	2	0	5	0
AT1G23410	Ribosomal protein S27a / Ubiquitin family protein	0	0	3	4
AT1G32220	NAD(P)-binding rossmann-fold superfamily protein	2	0	5	0
AT1G53540	HSP20-like chaperones superfamily protein	0	0	7	0
AT2G07698	ATPase, F1 complex, alpha subunit protein	0	3	2	2
AT2G21390	Coatomer, alpha subunit	0	3	0	4
AT2G33150	3-Ketoacyl-coa thiolase 2, KAT2	4	3	0	0
AT2G36530	Low expression of osmoticially responsive genes 2, LOS2	0	4	3	0
AT3G14990	DJ-1 homolog A, DJ-1A	0	3	4	0
AT3G17810	Pyrimidine 1, PYD1	2	0	3	2
AT5G10450	G-BOX regulating factor 6, GRF6	0	7	0	0
AT5G14040	Mitochondrial phsphate transporter 3, MPT3	0	2	3	2
AT5G24300	Starch synthase 1, SS1	7	0	0	0
AT5G64040	Encodes the only subunit of photosystem I, PSAN	0	2	2	3
AT1G09620	Leucine-tRNA ligases	0	4	0	2
AT1G22780	Pointed first leaves 1, PFL1	0	4	2	0
AT1G59960	NAD(P)-linked oxidoreductase superfamily protein	2	0	2	2

AT1G67700	Hypersensitive to high light 1, HHL1	2	0	2	2
AT1G68830	STT7 homolog STN7, STN7	6	0	0	0
AT1G76080	Chloroplastic drought-induced stress protein of 32 KD, CDSP32	4	0	2	0
AT2G30970	Aspartate aminotransferase 1, ASP1	0	6	0	0
AT2G33040	Gamma subunit of MT ATP synthase, ATP3	0	3	3	0
AT2G35410	RNA-binding (RRM/RBD/RNP motifs) family protein	2	4	0	0
AT3G02090	Mppbeta	0	4	0	2
AT3G07040	Resistance to *P. SYRINGAE* pv Maculicola 1, RPM1	3	3	0	0
AT3G11510	Ribosomal protein S11 family protein	3	3	0	0
AT3G27690	Light-harvesting chlorophyll B-binding 2, LHCB2	0	0	3	3
AT3G46780	Plastid transcriptionally active 16, PTAC16	0	4	2	0
AT3G52180	Dual-specificity protein phosphatase 4, DSP4	3	0	3	0
AT3G58510	DEA(D/H)-box RNA helicase family protein;	0	3	3	0
AT4G02770	Photosystem I subunit D-1, PSAD-1	3	0	0	3
AT4G09010	Ascorbate peroxidase 4, APX4	0	4	0	2
AT4G27450	Aluminium induced protein with YGL and LRDR motifs	0	4	0	2
AT4G35250	High chlorophyll fluorescence phenotype 244, HCF244	2	4	0	0
AT4G37910	Mitochondrial heat shock protein 70-1, MTHSC70-1	4	0	0	2
AT5G10540	Thimet metalloendopeptidase 2, TOP2	0	4	2	0
AT5G15450	Albino and pale green 6, APG6	0	2	2	2
AT5G20630	Germin-like protein 3, GLP3	6	0	0	0
AT5G54270	Light-harvesting chlorophyll B-binding protein 3, LHCB3	0	0	3	3
ATCG00750	Ribosomal protein S11, RPS11	0	2	2	2
ATCG01110	NAD(P)H dehydrogenase subunit H, NDHH	0	3	3	0
AT1G02560	Nuclear encoded clp protease 5, NUCLEAR CLPP 5	0	3	2	0
AT1G05010	Ethylene forming enzyme, EFE	0	0	3	2
AT1G07660	Histone superfamily protein	2	0	0	3
AT1G18060	Unknown protein	2	0	3	0
AT1G24180	IAA-conjugate-resistant 4, IAR4	3	0	2	0
AT1G66430	Pfk B-like carbohydrate kinase family protein	2	3	0	0
AT1G74470	Encodes for a multifunctional protein with geranylgeranyl reductase activity	3	2	0	0
AT2G16950	Transportin 1, TRN1	0	5	0	0
AT2G27680	NAD(P)-linked oxidoreductase superfamily protein	0	3	2	0
AT2G36160	Ribosomal protein S11 family protein	3	0	0	2
AT2G43090	Aconitase/3-isopropylmalate dehydratase protein	5	0	0	0

AT3G04790	Embryo defective 3119, EMB3119	2	3	0	0
AT3G09350	Hsp70-binding protein 1, HspBP-1	0	5	0	0
AT3G23400	Fibrlillin 4, FIB4	0	0	2	3
AT3G44310	Nitrilase 1	0	5	0	0
AT1G60190	PUB19	0	0	0	3
AT5G03940	Chloroplast signal recognition particle 54 KDA subunit, 54CP	3	0	0	2
AT5G14780	Fornate dehydrogenase, FDH	0	3	0	2
AT5G19780	Tubulin alpha-5, TUA5	0	5	0	0
AT5G19820	Embryo defective 2734, EMB2734	0	2	3	0
AT5G43940	Alcohol dehydrogenase 2, ADH2	0	3	2	0
AT5G45170	Haloacid dehalogenase-like hydrolase (HAD) superfamily protein	2	0	3	0
ATMG01190	ATP synthase subunit 1, ATP1	0	0	5	0
AT1G01560	MAP kinase 11, ATMPK11	0	0	4	0
AT1G01800	NAD(P)-binding Rossmann-fold superfamily protein	0	0	2	2
AT1G12840	Vacuolar ATP synthase subunit C, VHA-C	0	2	2	0
AT1G24100	UDP-glucosyl transferase 74B1, UGT74B1	2	0	0	2
AT1G32900	Granule bound starch synthase 1, GBSS1	1	0	3	0
AT1G55480	Protein containing PDZ domain	0	4	0	0
AT1G55490	Chaperonin 60 beta, CPN60B	2	2	0	0
AT1G56050	GTP-binding protein-related	0	2	0	2
AT1G56190	Phosphoglycerate kinase family protein	4	0	0	0
AT1G78380	Glutathione S-transferase TAU 19, GSTU19	0	0	2	2
AT2G01350	Quinolinate phoshoribosyl transferase, QPT	0	0	2	2
AT2G26080	Glycine decarboxylase P-protein 2, GLDP2	0	4	0	0
AT2G30860	Glutathione S-transferase phi 9, GSTF9	0	4	0	0
AT2G38810	Histone H2A 8, HTA8	0	0	2	2
AT2G41100	Calmodulin LIKE 4, CAL4	2	0	2	0
AT2G42520	P-loop containing nucleoside triphosphate hydrolases superfamily protein	2	0	0	2
AT3G08590	2,3-Biphosphoglycerate-independent phosphoglycerate mutase 2, IPGAM2	0	2	2	0
AT3G15020	Mitochondrial malate dehydrogenase 2, MMDH2	0	0	0	4
AT3G22520	Unknown protein	2	2	0	0
AT3G46970	Alpha-glucan phosphorylase 2, PHS2	0	0	2	2
AT3G52930	Fructose-bisphosphate aldolase 8, FBA8	0	1	0	3
AT3G55410	2-Oxoglutarate dehydrogenase	2	2	0	0

AT3g55800	Sedoheptulose-bisphosphatase, SBPASE	4	0	0	0
AT3G58610	Ketol-acid reductoisomerase	2	2	0	0
AT4G09000	General regulatory factor 1, GRF1	0	4	0	0
AT4G09650	ATP synthase delta-subunit gene, ATPD	2	2	0	0
AT4G14160	Sec23/Sec24 protein transport family protein;	0	0	2	2
AT4G20890	Tubulin beta-9 chain, TUB9	4	0	0	0
AT4G26530	Fructose-bisphosphate aldolase 5, FBA5	0	0	4	0
AT4G29130	Glucose insensitive 2, GIN2	0	4	0	0
AT4G30920	Leucyl aminopeptidase, LAP2	4	0	0	0
AT4G35630	Phosphoserine aminotransferase, PSAT	0	2	2	0
AT4G38460	Geranylgeranyl reductase, GGR	2	0	2	0
AT5G01750	Protein of unknown function	0	0	2	2
AT5G30510	Plastid ribosomal protein S1, PRPS1	0	2	2	0
AT5G46110	Acclimation of photosynthesis to environment 2, APE2	2	2	0	0
AT5G46290	Ketoacyl-ACP synthase 1, KAS1	0	2	2	0
AT5G50920	De-regulated cao accumulation 1, DCA1	0	0	0	4
AT5G50920	Heat shock protein 93-V, HSP93-V	4	0	0	0
AT5G51070	Early responsive to dehydration 1, ERD1	4	0	0	0
AT5G51440	HSP20-like chaperones superfamily protein	4	0	0	0
AT5G51820	Phosphoglucomutase, PGM	4	0	0	0
AT5G55280	Homolog of bacterial cytokinesis Z-ring protein FTSZ 1-1, FTSZ1-1,	2	0	0	2
AT5G62690	Tubulin beta chain 2, TUB2	0	4	0	0
AT5G64050	Ovule abortion 3, OVA3	4	0	0	0
ATCG00770	Ribosomal protein S8, RPS8	0	0	2	2
ATCG00800	Encodes a chloroplast ribosomal protein S3	0	0	0	4
AT1G03160	FZO-LIKE, FZL	3	0	0	0
AT1G04410	Cytosolic-NAD-dependent malate dehydrogenase 1, C-NAD-MDH1	0	0	3	0
AT1G06430	Ftsh protease 8, FTSH8	0	0	3	0
AT1G22940	Thiamine requiring 1, TH1	3	0	0	0
AT1G24610	Rubisco methyltransferase family protein	3	0	0	0
AT1G30580	GTP binding	0	0	3	0
AT1G49750	Leucine-rich repeat (LRR) family protein	3	0	0	0
AT1G55450	S-adenosyl-L-methionine-dependent methyltransferases superfamily protein	0	0	3	0
AT1G55850	Cellulose synthase like E1, CSLE1	0	0	3	0

AT1G70820	Phosphoglucomutase	0	0	3	0
AT1G78850	Curculin-like (mannose-binding) lectin family protein	3	0	0	0
AT2G19590	ACC oxidase 1, ACO1	0	3	0	0
AT2G21370	XYLULOSE KINASE 1, XK1	0	0	3	0
AT2G35660	CTF2A	3	0	0	0
AT2G40590	Ribosomal protein S26e family protein;	0	0	0	3
AT2G41730	Unknown protein	0	3	0	0
AT2G41790	Insulinase (Peptidase family M16) family protein	0	0	0	3
AT2G44120	Ribosomal protein L30/L7 family protein	0	0	0	3
AT3G04880	DNA-damage-repair/toleration 2, DRT102	0	0	0	3
AT3G05560	Ribosomal L22e protein family	0	3	0	0
AT3G06050	Peroxiredoxin IIF, PRXIIF	3	0	0	0
AT3G08920	Rhodanese/Cell cycle control phosphatase superfamily protein	1	0	2	0
AT3G09940	Monodehydroascorbate reductase 3, MDAR3	0	0	0	3
AT3G10130	SOUL heme-binding family protein	3	0	0	0
AT3G16640	Translationally controlled tumor protein, TCTP	0	3	0	0
AT3G43300	Bfa-visualized endocytic trafficking defective 1, BEN1	0	0	0	3
AT3G44860	Farnesoic acid carboxyl-O-methyltranseferase, FAMT	0	0	3	0
AT3G56150	Eukaryotic translation initiation factor 3C, EIF3C	0	3	0	0
AT4G00490	Beta-amylase 2, BAM2	1	2	0	0
AT4G01690	Encodes protoporphyrinogen oxidase, PPOX	3	0	0	0
AT4G16760	Acyl-coa oxidase 1, ACX1	0	3	0	0
AT4G22010	SKU5 similar 4, SKS4	3	0	0	0
AT4G24800	EIN2 C-terminus interacting protein 1, ECIP1	0	3	0	0
AT4G39970	Haloacid dehalogenase-like hydrolase (HAD) superfamily protein	3	0	0	0
AT4G39980	3-Deoxy-D-arabino-heptulosonate 7-phosphate synthase 1, DHS1	0	0	3	0
AT5G02570	Histone superfamily protein	0	3	0	0
AT5G17380	Thiamine pyrophosphate dependent pyruvate decarboxylase family protein	0	0	0	3
AT5G41670	6-Phosphogluconate dehydrogenase family protein	0	0	3	0
AT5G44720	Molybdenum cofactor sulfurase family protein;	0	0	0	3
AT5G50920	Clpc homologue 1, CLPC1	0	3	0	0
AT5G53480	Homolog of human KPNB1	0	0	0	3
AT5G59290	UDP-glucuronic acid decarboxylase 3, UXS3	0	3	0	0
ATCG00160	Ribosomal protein S2, RPS2	3	0	0	0

ATCG00500	Acetyl-coa carboxylase carboxyl transferase subunit beta, ACCD	3	0	0	0
AT1G04420	NAD(P)-linked oxidoreductase superfamily protein	0	0	2	0
AT1G08520	Pigment defective embry 166, PDE166	2	0	0	0
AT1G09340	Heteroglycan-interacting protein 1.3, HIP1.3	0	0	0	2
AT1G16720	High chlorophyll fluorescence phenotype 173, HCF173	2	0	0	0
AT1G17290	Alanine aminotransferas 1, ALAAT1	0	2	0	0
AT1G23130	Polyketide cyclase/dehydrase and lipid transport superfamily protein	2	0	0	0
AT1G27020	Unknown protein	2	0	0	0
AT1G33120	Ribosomal protein L6 family	0	2	0	0
AT1G33360	Encodes ClpX3, a subunit of the Clp protease complex	2	0	0	0
AT1G53280	DJ-1 homolog B, DJ-1B	2	0	0	0
AT1G54270	Member of eIF4A - eukaryotic initiation factor 4A, EIF4A-2	0	0	2	0
AT1G62020	Coatomer, alpha subunit	0	0	2	0
AT1G62780	Unknown protein;	2	0	0	0
AT1G67430	Ribosomal protein L22p/L17e family protein	0	2	0	0
AT1G71330	Non-intrinsic ABC protein 5, NAP5	0	0	2	0
AT1G72160	Sec14p-like phosphatidylinositol transfer family protein	0	0	0	2
AT1G74060	Ribosomal protein L6 family protein	0	0	2	0
AT1G75780	Tubulin beta-1 chain, TUB1	0	0	0	2
AT1G76680	12-Oxophytodienoate reductase 1, OPR1	0	2	0	0
AT2G17020	F-box/RNI-like superfamily protein	0	2	0	0
AT2G17360	Ribosomal protein S4 (RPS4A) family protein;	0	0	0	2
AT2G18450	Succinate dehydrogenase 1-2, SDH1-2	0	2	0	0
AT2G21385	Unknown protein	2	0	0	0
AT2G41740	VLN2	0	0	2	0
AT2G43910	Harmless to ozone later 1, HOL1	0	2	0	0
AT2G44160	Methylenetetrahydrofolate reductase 2, MTHFR2	0	0	0	2
AT3G01510	Lile SEX4 1, LSF1	2	0	0	0
AT3G02530	TCP-1/cpn60 chaperonin family protein	0	0	0	2
AT3G04550	Unknown protein	2	0	0	0
AT3G04870	Pigment defective embryo 181, PDE181	2	0	0	0
AT3G06510	Sensitive to freezing 2, SFR2	0	2	0	0
AT3G09200	Ribosomal protein L10 family protein	0	2	0	0
AT3G10090	Nucleic acid-binding, OB-fold-like protein	0	2	0	0
AT3G11910	Ubiquitin-specific protease 13, UBP13	2	0	0	0

AT3G11940	Arabidopsis minute-like 1, AML1	0	2	0	0
AT3G20680	Unknown protein	2	0	0	0
AT3G22200	Hexenal response1, HER1	0	0	0	2
AT3G48410	Alpha/beta-hydrolases superfamily protein	0	2	0	0
AT3G50950	Hopz-activated resistance 1, ZAR1	2	0	0	0
AT3G53870	Ribosomal protein S3 family protein	0	0	2	0
AT3G55250	Pigment defective 329, PDE329	2	0	0	0
AT3G55610	Delta 1-pyrroline-5-carboxylate synthase 2, P5CS2	0	0	0	2
AT3G59890	Dihydrodipicolinate reductase, bacterial/plant	0	0	2	0
AT3G62530	ARM repeat superfamily protein	2	0	0	0
AT4G01370	MAP kinase 4, MAPK4	0	2	0	0
AT4G01870	TolB protein-related	0	2	0	0
AT4G05160	Encodes a peroxisomal protein	0	0	2	0
AT4G11380	Adaptin family protein	0	0	2	0
AT4G13010	Oxidoreductase, zinc-binding dehydrogenase family protein	2	0	0	0
AT4G14960	Tubulin alpha-6, TUA6	0	0	0	2
AT4G15110	Cytochrome P450	0	2	0	0
AT4G24220	Vein patterning 1, VEP1	0	2	0	0
AT4G24820	26S Proteasome, regulatory subunit Rpn7	0	2	0	0
AT4G26300	Embryo defective 1027, EMB1027	0	2	0	0
AT4G26900	HIS HF	2	0	0	0
AT4G27640	ARM repeat superfamily protein	0	0	0	2
AT4G29060	Embryo defective 2726, EMB2726	2	0	0	0
AT4G29840	Methionine over-accumulator 2, MTO2	0	2	0	0
AT4G34670	Ribosomal protein S3Ae	0	2	0	0
AT4G36810	Geranyl diphosphate synthase 11, GGPPS11	2	0	0	0
AT4G39520	Encodes a member of the DRG (developmentally regulated G-protein) family	0	2	0	0
AT5G03340	Cell division cycle 48C, CDC48C	0	0	0	2
AT5G09590	Heat shock cognate, HSC70-5	2	0	0	0
AT5G09810	Member of Actin gene family	0	0	2	0
AT5G13420	Transaldolase 2, TRA2	0	2	0	0
AT5G14930	Senescence-associated gene 101, SAG101	2	0	0	0
AT5G15210	Homeobox protein 30, HB30	2	0	0	0
AT5G16370	Acyl activating enzyme 5, AAE5	2	0	0	0
AT5G17310	UDP-glucose pyrophosphorylase 2, UGP2	0	2	0	0

AT5G17990	Phosphoribosylanthranilate transferase 1, PAT1	0	0	2	0
AT5G18380	Ribosomal protein S5 domain 2-like superfamily protein	0	2	0	0
AT5G19770	Tubulin alpha-3, TUA3	0	2	0	0
AT5G20010	Ras-related nuclear protein, RAN1	0	0	0	2
AT5G23060	Calcium sensing receptor, CAS	0	2	0	0
AT5G26000	Thioglucoside glucohydrolase 1, TGG1	0	2	0	0
AT5G26860	Lon protease 1, LON1	2	0	0	0
AT5G27540	Miro-related GTPase 1, MIRO1	0	2	0	0
AT5G35530	Ribosomal protein S3 family protein	0	2	0	0
AT5G36210	Alpha/beta-Hydrolases superfamily protein	0	2	0	0
AT5G36230	ARM repeat superfamily protein	0	0	0	2
AT5G37600	Glutamine synthase clone R1, GSR 1	0	0	0	2
At5g42310	Pentatricopeptide repeat (PPR-like) superfamily protein	2	0	0	0
AT5G43780	APS4	0	0	2	0
AT5G44020	HAD superfamily	2	0	0	0
AT5G45390	CLP Protease P4, CLPP4	2	0	0	0
AT5G46290	3-Ketoacyl-acyl carrier protein synthase 1, KAS1	0	0	0	2
AT5G48300	ADP glucose pyrophosphorylase 1, ADG1	2	0	0	0
AT5G48570	ATFKBP65	0	0	0	2
AT5G48570	Encodes one of the 36 carboxylate clamp (CC)-tetratricopeptide repeat (TPR) proteins	0	2	0	0
AT5G48960	HAD-superfamily hydrolase	2	0	0	0
AT5G50850	Macci-bou, MAB1	2		0	0
AT5G58710	Rotamase CYP 7, ROC7	2	0	0	0
AT5G61780	Tudor-SN protein 2, TUDOR2	0	2	0	0
AT5G65620	Organellar oligopeptidase, OOP	2	0	0	0
ATMG00160	Cytochrome oxidase 2, COX2	0	2	0	0
ATMG00510	NADH dehydrogenase subunit 7, NAD7	2	0	0	0
AT2G01250	Ribosomal protein L30/L7 family protein	0	1	0	0
AT5G35621	Eukaryotic initiation factor (ISO)5E, EIFISO4E	0	0	1	0

*Normalized peptide number is the peptide number detected by shortgun MS/MS from the amount of insoluble proteins isolated from 0.1 mg total protein for each genotype. Among the ~170 most abundant proteins detected, those with ≥2 fold enrichment in the *chip* mutant are highlighted in bright green, while those with ≥2 fold enrichment in the *nbr1* mutant are highlighted in yellow
