# Supplementary material for: E3 Ubiquitin Ligase CHIP and NBR1-Mediated Selective Autophagy Protect Additively against Proteotoxicity in Plant Stress Responses
Source: PLoS Genet. 2014 Jan 30;10(1):e1004116. doi: 10.1371/journal.pgen.1004116 (PMC3907298; doi:10.1371/journal.pgen.1004116)
Supplement: Table S3 — Primers for identifying the T-DNA insertions. (PDF) [file pgen.1004116.s006.pdf]

Table S3: Primers for identifying the T-DNA insertions

Mutant	Name	Forward	Reverse
chip-1	Salk_048371	GGAAACTTGTGAGGCTGCTC	GCCACTGCCTCTTTGATAGC
chip-2	Salk_059253	GGAAACTTGTGAGGCTGCTC	GCCACTGCCTCTTTGATAGC
cat2-1	Salk_076998	GGGCAGGTCCTATCCTTCTT	GAACCATCCATGTGCCTGTA
cat3-1	GABI_110C11	CATCGAACAAAAGGCTTGAA	TCAGGGTTACGATACCTCTTTCTC
