# Supplementary material for: E3 Ubiquitin Ligase CHIP and NBR1-Mediated Selective Autophagy Protect Additively against Proteotoxicity in Plant Stress Responses
Source: PLoS Genet. 2014 Jan 30;10(1):e1004116. doi: 10.1371/journal.pgen.1004116 (PMC3907298; doi:10.1371/journal.pgen.1004116)
Supplement: Table S4 — Primers for qRT-PCR. (PDF) [file pgen.1004116.s007.pdf]

Table S4: Primers for qRT-PCR

Gene	GI	Forward	Reverse
CHIP	AT3G07370	CGGTCATGGGAACTGAATAG	TGGCACCTCAGTTGGTTTAT
Actin2	AT3G18780	GCTGACCGTATGAGCAAAGA	ATCTGCTGGAATGTGCTGAG
ATG5	AT5G17290	ATGGGAACAGTCGAAGATGA	ACGAGATGTCATCCCAGGTA
ATG6	AT3G61710	AGCCATTGTGCTTAGAATGC	CCTTCTAACCTCTGAACGCA
ATG7	AT5G45900	TCTATGACCCGTGTCACCTT	GAGGCTTGACCAACAAGAGA
ATG8a	AT4G21980	CAAGCTTGGAGCTGAGAAAG	GCAACGGTAAGAGATCCAAA
ATG9	AT2G31260	TGGGAAGAGAATGCAAGAAG	ACCGTAATGTGGTGCTTGAT
ATG10	AT3G07525	CATGGTTCAAGCTACATCCC	GGGATCCTAAGACCAACCAC
ATG18a	AT3G62770	TGTTTCTCAGGGTGTTGGTT	TGAGAGCGAAGCAAGCTATT
NBR1	AT4G24690	GAGGAAATGGGTTTCAAGGA	ATTGGATCCCACTCGCTAAC
CAT2	AT4G35090	TGATAAGCTGCTTCAAACCC	TGACGAACCTGGTCATACCT
CAT3	AT1G20620	GACTACATGTCCCACTTGCC	TCTTGATCCCACAAGTTGGT
